# A toolbox for microvalve-based bioprinting

**DOI:** 10.1088/1758-5090/ae54d1

**Published:** 2026-04-02

**Authors:** I Deniz Derman, Medine Dogan Sarikaya, Yasar Ozer Yilmaz, Deepak Gupta, Syed Hasan Askari Rizvi, Taino Rivera, Ibrahim T Ozbolat

**Affiliations:** 1 Engineering Science and Mechanics Department, Penn State University, University Park, PA 16802, United States of America; 2 The Huck Institutes of Life Sciences, Penn State University, University Park, PA 16802, United States of America; 3 Genome and Stem Cell Center (GENKOK), Erciyes University, Kayseri, Türkiye; 4 Department of Nanoscience and Nanoengineering, Istanbul Technical University, Istanbul, Türkiye; 5 Biomedical Engineering Department, Penn State University, University Park, PA 16802, United States of America; 6 Materials Research Institute, Penn State University, University Park, PA 16802, United States of America; 7 Cancer Institute, Penn State University, University Park, PA 16802, United States of America; 8 Neurosurgery Department, Penn State University, University Park, PA 16802, United States of America

**Keywords:** droplet, bioprinting, micro-valve, dimensionless numbers, ligament dynamics

## Abstract

Microvalve-based bioprinting (MBB) enables precise deposition of bioinks in the form of droplets through the controlled ejection of nanoliter-scale cylindrical ligaments. Despite its increasing use in tissue biofabrication, standardization criteria for assessing bioink printability remain limited. In this study, we present a quantitative printability toolbox for evaluating various bioinks, including fibrinogen, collagen type I, Matrigel, alginate, agarose and methacrylated gelatin (GelMA), in the context of MBB. We systematically analyzed how rheological properties and the contact angle influence ligament formation and droplet ejection. High-speed imaging captured ligament dynamics such as velocity and volume as well as droplet-substrate interactions. The role of Tween 20 (T20) surfactant was further investigated to reduce interfacial aggregation and improve droplet uniformity. Our results revealed viscosity and concentration thresholds specific to each bioink, enabling the construction of a comprehensive printability map correlating bioink properties with ligament stability and droplet printability. This framework provides a practical guide for bioink optimization in MBB towards reproducible fabrication of complex biological structures for biomedical applications.

## 1. Introduction

Droplet-based bioprinting (DBB) is a high-resolution, non-contact biofabrication approach that enables the spatially controlled, on-demand deposition of bioinks and living cells in discrete volumes, facilitating the fabrication of 3D biological constructs with cell-level precision [1]. Unlike extrusion-based methods, DBB achieves finer control of feature size and spatial organization through layer-by-layer accumulation of droplets, which is critical for replicating complex tissue morphologies and functional interfaces. Among DBB techniques, microvalve-based bioprinting (MBB) has been considered a versatile, scalable, and broadly compatible modality. MBB utilizes pneumatic actuation and microvalve control to dispense nanoliter-scale droplets at moderate to high viscosities (up to ∼70 mPa s), attaining feature sizes on the order of 100–300 *μ*m [2, 3]. The platform’s tunable actuation frequency (up to ∼1 kHz), low-pressure operation, and minimal heat generation ensure high-throughput bioprinting while preserving cell viability, which are essential attributes for demanding applications, such as engineered tissues, organoids, and personalized medicine. Furthermore, MBB accommodates a wide range of bioinks, including fibrinogen, collagen, Matrigel, and alginate [4, 5], some of which are challenging in other DBB modalities, such as inkjet bioprinting.

Despite its technological promise, MBB faces several persistent engineering barriers that constrain printability and reproducibility. The small nozzle orifice (100–250 *μ*m) is prone to clogging, especially with higher or viscoelastic protein-based and polymeric solutions. Surface adsorption, air-liquid, and solid-liquid interface dynamics further promote undesired aggregation, precipitation, or phase separation, which can disrupt droplet ejection and compromise construct integrity [2, 3]. Additionally, cell settling and sedimentation in the bioink reservoir can lead to heterogeneity in cell distribution, reducing reproducibility and uniformity of bioprinted constructs [6–8]. The non-spherical, ligament-shaped deposits—a signature of MBB—exhibit elevated inertial impact energy upon substrate contact, increasing vulnerability to splashing, breakup, and satellite droplet formation, all of which may impair resolution and defect-free patterning [9–11]. Stable droplet formation demands precise management of key jetting parameters, such as valve opening time, pressure, and Ohnesorge number (*Oh*), where *Z* should be maintained within the printable range (typically 1 ⩽ *Z* ⩽ 10), thereby preventing satellite droplets and ensuring consistent printing fidelity [1, 5].

This study focuses on six representative bioinks, including fibrinogen, collagen type I, Matrigel, alginate, agarose and methacrylated gelatin (GelMA) chosen for their extensive utility in 3D bioprinting, proven biocompatibility, and distinct gelation mechanisms. Collagen type I provides essential extracellular matrix (ECM) structure and integrin-binding sites; fibrinogen undergoes rapid enzymatic conversion into fibrin during *in-situ* gelation; Matrigel is a basement membrane analog rich in laminin, collagen IV, and growth factors, supporting cell differentiation and migration; alginate yields ionically crosslinkable hydrogels with tunable mechanical stiffness and cytocompatibility; agarose, a highly hydrated and biocompatible polysaccharide, mimics key ECM properties and exhibits thermoreversible gelation in response to temperature changes; GelMA, a derivative of gelatin, retains the arginine-glycine-aspartic acid cell-binding motif of collagen and combines high biocompatibility with light-mediated crosslinking capability [12–18]. Their broad rheological profiles and gelation behaviors render them ideal candidates for a systematic investigation of MBB printability thresholds and establishing formulation principles.

Here, a quantitative printability toolbox is established for MBB, incorporating rheological metrics, jetting dynamics, and substrate interaction parameters to predict and optimize bioprinting outcomes across diverse bioinks. By characterizing viscosity, concentration, ligament volume and velocity, and contact angle, and their effects on stable droplet formulation, this framework delivers experimentally validated criteria for the rational design of bioink formulations and bioprinting protocols. The proposed toolbox serves both as an empirical reference for reproducible, high-fidelity MBB, and as a foundation for further innovation in tissue engineering, drug screening, and disease modeling.

## 2. Materials and methods

### 2.1. Bioink preparation

#### 2.1.1. Fibrinogen

Fibrinogen from bovine plasma (Sigma-Aldrich) was dissolved in Dulbecco’s phosphate-buffered saline (DPBS, Corning, 1X) to prepare a 100 mg ml^−1^ stock solution. The stock was aliquoted and stored at −20 °C until further use to prevent degradation. The stock solution was thawed on ice and diluted to the desired concentrations of 5, 10, 15, and 20 mg ml^−1^ using cold DPBS. All dilutions were performed under aseptic conditions and solutions were maintained at 4 °C during rheological and printability characterization to minimize degradation and aggregation. The solutions were filtered through a 0.22 *μ*m syringe filter to remove particulates before use. For surfactant-containing formulations, T20 (Sigma-Aldrich) was added at ⩽0.001% v/v to reduce surface tension (*σ*), enhance droplet formation, and avoid cytotoxicity, verified by cell viability assays [19, 20].

#### 2.1.2. Collagen type I

Collagen type I was purchased (rat tail; 11 mg ml^−1^ in 0.02 N acetic acid, Corning) and stored at 4 °C until use. Prior to each experiment, the collagen solution was neutralized to the desired concentrations (1, 3, 5, and 7 mg ml^−1^) using DPBS (10X), 1 N NaOH (Sigma-Aldrich), and deionized (DI) water under sterile conditions. Neutralized solutions were maintained at 4 °C to prevent premature gelation. The solutions were filtered through a 0.22 *μ*m syringe filter to remove particulates before use. Surfactant T20 (Sigma-Aldrich) was added at ⩽0.001% v/v in relevant experiments, ensuring minimal interference with protein structure and preserving biocompatibility.

#### 2.1.3. Matrigel

Matrigel growth factor reduced basement membrane matrix, phenol red-free, LDEV-free (Corning) was used as received and handled on ice to maintain its liquid state. Matrigel matrix was diluted with cold DPBS (1X) to final concentrations of 0.1%, 1%, 5%, and 10% (v/v). All procedures were conducted using pre-chilled pipettes and tubes to prevent spontaneous gelation. The solutions were filtered through a 0.22 *μ*m syringe filter to remove particulates and sterilize the material before use. T20 was added at ⩽0.001% v/v to all Matrigel formulations to minimize *σ* and ensure consistent droplet formation during bioprinting.

#### 2.1.4. Alginate

Alginic acid sodium salt from brown algae (Sigma-Aldrich) was dissolved in DPBS (1X) to prepare stock solutions. Alginate was then diluted to final concentrations of 0.05, 0.1, 0.25, and 0.5% (w/v) and stirred at room temperature (RT) for at least 4 h to ensure complete dissolution. The solutions were filtered through a 0.22 *μ*m syringe filter to remove particulates before use. T20 was incorporated at ⩽0.001% v/v, where applicable to improve droplet formation without introducing cytotoxic effects.

#### 2.1.5. Agarose

Low-gelling-temperature agarose (Sigma-Aldrich) was dissolved in DPBS (1X) to prepare stock solutions by heating at 90 °C with continuous stirring until complete dissolution. The agarose solution was then allowed to cool to 50 °C and diluted to final concentrations of 0.1, 0.3, 0.5, and 0.7% (w/v) using pre-warmed DPBS. All solutions were gently mixed to avoid bubble formation and maintained at 40 °C prior to bioprinting to prevent premature gelation. The prepared agarose bioink was used immediately for rheological and printability characterization. T20 was added at ⩽0.001% v/v, a concentration selected to improve bioprinting stability while preserving biocompatibility.

#### 2.1.6. GelMA

GelMA was synthesized following a previously reported protocol [21] by reacting methacrylic anhydride with gelatin from porcine skin (Sigma-Aldrich), followed by purification using a 12–14 kDa molecular weight cut-off dialysis membrane and subsequent freeze drying. The degree of methacrylation was approximately 80%. For bioink preparation, GelMA was dissolved in DPBS at 50 °C under constant stirring to obtain homogeneous stock solutions. The freeze-dried GelMA was diluted to final concentrations of 0.5, 1, 3, and 5% (w/v) and maintained at 40 °C to prevent physical gelation prior to bioprinting. For photocrosslinkable formulations, lithium phenyl-2,4,6-trimethylbenzoylphosphinate (Sigma-Aldrich) was added at a final concentration of 0.5% (w/v) and mixed thoroughly under light-protected conditions. All GelMA solutions were prepared under sterile conditions and used immediately after preparation to ensure consistent rheological behavior. T20 was incorporated at ⩽0.001% v/v to reduce surface tension and enhance extrusion fidelity without compromising cytocompatibility. GelMA solutions were exposed to UV light for 10 s to induce crosslinking prior to the LIVE/DEAD assay.

### 2.2. Rheological measurements

**Figure 1.**
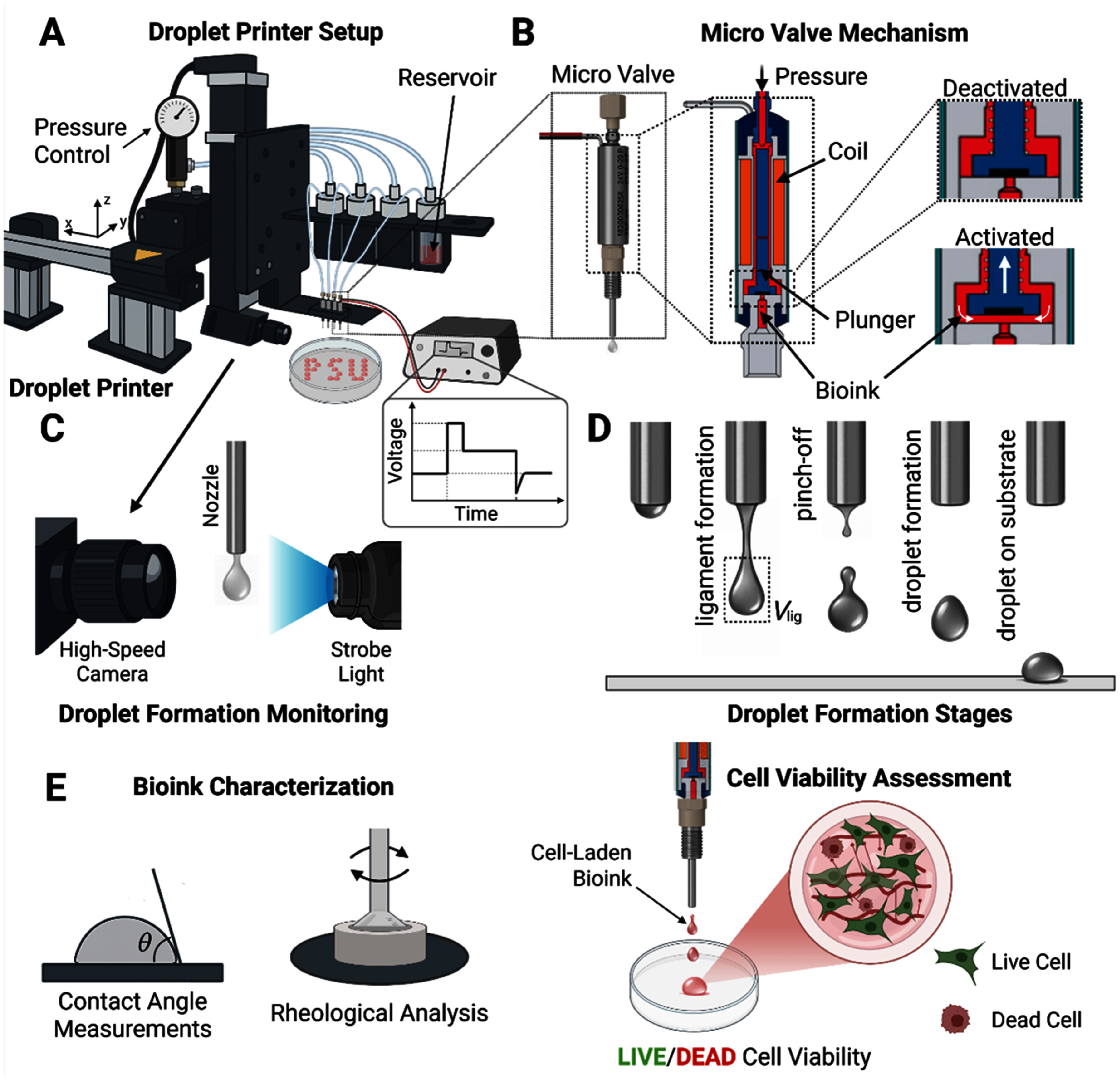
Schematic overview of the MBB setup, operational mechanism, and bioink characterization workflow. (A) Custom microvalve droplet bioprinter equipped with a pressure control unit, multiple reservoirs, and a motorized *XYZ* positioning system for precise droplet deposition, and the square wave voltage pulse modulates nozzle actuation timing. (B) Microvalve design and actuation principle, showing magnetic coil-driven plunger- operation and the transition from a deactivated state to an activated state for droplet ejection. (C) Integration of a high-speed camera and strobe illumination for real-time visualization and analysis of droplet formation dynamics. (D) Sequential physical stages of droplet formation, including ligament formation, pinch-off, droplet formation, and deposition on the substrate (the ligament volume is indicated as *V*_lig_). (E) Bioink characterization and post-bioprinting assessment, including contact angle measurements, rheological analysis, and LIVE/DEAD assay for evaluating cell viability within bioprinted constructs.

Bulk rheological properties of all bioinks were measured using a stress-controlled rheometer (Anton Paar MCR 302) equipped with a cone-plate measuring system (CP25-1; 25 mm diameter, 1° cone angle, 103 *µ*m truncation gap) (figure 1(E)). Prior to measurements, the instrument was calibrated according to the manufacturer’s protocol. Due to the temperature sensitivity of fibrinogen, collagen type I, and Matrigel, all such samples were kept on ice prior to measurement, except for agarose, which was kept at 40 °C and loaded immediately to prevent their premature gelation. Rheological measurements for fibrinogen, collagen type I, and Matrigel were conducted at 4 ± 0.5 °C, while agarose measurements were conducted at 40 °C using the integrated Peltier temperature control, whereas alginate samples were analyzed at RT.

For each sample, 200 *µ*l of bioink solution was carefully pipetted onto the rheometer stage and pre-sheared at 60 s^–1^ for 1 min to remove shear history effects related to sample loading. Steady-shear flow curves were obtained by sweeping the shear rate from 0 to 1000 s^–1^ followed by a reverse sweep from 1000 to 0 s^–1^. Dynamic mechanical analysis was performed through strain amplitude sweeps at a constant angular frequency of 1 rad s^−1^ to determine the storage (G′) and loss modulus (*G″*) within the linear viscoelastic region. At least three independent samples were tested for each concentration, and the reported values represent mean ± standard deviation (SD). Between each sample, the cone and plate surfaces were thoroughly cleaned and dried to avoid cross-contamination.

### 2.3. MBB

Bioprinting experiments were performed using a custom-built bioprinter (MicroFab Technologies, jetlab 4), housed inside a vertical laminar flow cabinet (Air Science, Purair) to maintain sterile conditions. The setup was equipped with four independently controlled micro-valve dispensing devices (the Lee Company), each fitted with a replaceable nozzle with a 250 *µ*m orifice diameter (the Lee Company) (figure 1(A)).

Bioink solutions were loaded into dedicated fluid reservoirs and delivered to each microvalve under a regulated back pressure of ⩽800 mmHg, which was desired for stable droplet ejection (figure 1(B)). Droplet dispensing was actuated using a square-wave voltage pulse with a dwell voltage of 5 V and a dwell time of 1000 *μ*s. The valve opening and closing dynamics achieved rise and fall times of approximately 1 *μ*s, an echo voltage of 0 V, and an echo time of 20 *μ*s. During actuation, the electromagnetic microvalve was magnetized and opened for 1000 *μ*s, resulting in the ejection of cylindrical liquid jets (ligaments) (video S1). The system operated at a jetting frequency of 200 Hz to ensure precise deposition and minimize shear stress on cells.

Following each bioprinting session, microvalve nozzles were manually flushed with commercial bleach (Clorox) and sterile DI water to prevent clogging or cross-contamination. The cleaning process included manually holding each valve open with a handheld magnet, enabling full perfusion of the cleaning agents through the dispensing system. All cleaned components were dried before subsequent use to maintain sterility.

At the start of each session, and after any nozzle replacement, we calibrated the ejected volume by dispensing 1000 droplets into a pre-weighed microtube on an analytical balance and dividing the net mass by the pulse count to obtain per-drop volume (video S2). Calibration was repeated at three dwell times bracketing the operating point to verify linearity of volume versus pulse width, and sessions were accepted only if the coefficient of variation was ⩽5% and the linear fit yielded *R*^2^ ⩾ 0.99, after which dwell time and back pressure were fixed for bioprinting. Substrates were then leveled and brought into focus using a built-in *Z* probe and a coaxial camera, followed by a three-point registration routine using reference markers in which a 3 × 3 test array (1 mm spacing) was deposited, imaged, and compared to the programmed pattern (video S3).

### 2.4. Cell culture

Normal human dermal fibroblasts (NHDFs-Ad-Der; Lonza) were used in this study. Cells were cultured in Fibroblast Growth Medium-2 (FGM®-2, Lonza) supplemented with the corresponding BulletKit (Lonza) in a humidified 5% CO₂ incubator (Thermo Fisher) at 37 °C. The growth medium was refreshed every two days. To passage, confluent NHDFs were washed three times with DPBS and detached using 0.25% trypsin/EDTA (Sigma-Aldrich) for 5 min at 37 °C. Cell suspensions were then centrifuged at 1500 rpm for 5 min at RT, and the resulting pellets were resuspended in the desired bioink formulations to prepare cell-laden solutions for bioprinting.

All bioink components were sterile filtered (0.22 *µ*m) or supplied sterile, and all handling steps were carried out in a Class II biosafety cabinet using aseptic techniques. Bioinks were prepared on ice (for fibrinogen, collagen type I and Matrigel), in RT (for alginate) and at 40 °C (for agarose and GelMA) inside the biosafety cabinet and mixed with cells using sterile, low-retention pipette tips. Cell-laden bioinks were transferred into sterile microcentrifuge tubes and subsequently loaded into the bioprinter under aseptic conditions inside the biosafety cabinet. Prior to bioprinting, the bioprinter stage and fixtures were disinfected with 70% ethanol, sterile nozzles were used, and only pre-sterilized substrates were mounted for cell bioprinting to ensure sterility throughout the process.

### 2.5. Printability assessment

Ligament velocity and volume were quantitatively measured to assess the printability characteristics of each bioink. Velocity measurements were conducted using a horizontally mounted built-in USB camera (figure 1(C)). For each experimental condition, ligament formation was imaged at 2400 *μ*s post actuation, with sweep analysis performed by incrementally varying the actuation pulse from 0 to 1800 or 2,400 *μ*s in 50 *μ*s steps. The process was recorded using screen recording software. ImageJ (NIH) was used to analyze the displacement of ligaments over time and calculate their average velocity.

Ligament volume was determined by collecting droplets in bulk: 1000 droplets were ejected per burst and collected into 1.5 ml Eppendorf tubes (Thermo Scientific), with three independent repetitions for each bioink (figure 1(D)). The collected mass was measured using a high-precision analytical balance (Excellence Plus XP, Mettler-Toledo). The average volume of individual droplets was calculated by dividing the total measured mass by the number of droplets and normalizing it using the solution density (*ρ*). *ρ* for each protein solution was measured independently. 100 *µ*l of each solution was pipetted into three pre-weighed, empty Eppendorf tubes. The filled tubes were weighed, and *ρ* was calculated by dividing the net mass by the known volume. For ligament volume measurements, cells were also encapsulated in bioink solutions (at their lowest bioink concentration) at densities of 0.5, 1, and 3 × 10^6^ cells ml^−1^. All measurements were performed in triplicate.

Concentration ranges selected for each bioink were determined based on a preliminary printability screening study conducted prior to experiments. Specifically, concentrations were chosen to span the stable printability window of our MBB system, where consistent droplet formation and ligament breakup could be achieved without nozzle clogging or irregular deposition. Concentrations outside this range either failed to produce stable droplets or resulted in excessive spreading or jetting instabilities. Therefore, the final concentration ranges used in this study were selected primarily based on printability constraints of the MBB system, while also remaining within commonly reported concentration ranges in the literature.

In this study, ligament velocity and volume were selected as primary quantitative descriptors to assess the printability of bioinks under MBB conditions. These parameters directly reflect the dynamic droplet formation process, ligament velocity correlates with the initial kinetic energy imparted to the fluid during valve actuation, while ligament volume represents the actual amount of bioink ejected per pulse. Together, they enable a reproducible and quantifiable means to characterize whether a bioink formulation can consistently produce droplets within a desirable size and speed range, without splashing, satellite formation, or valve clogging. These metrics are especially useful in establishing the operational window for printable regimes and were used to construct a predictive map of successful jetting conditions, serving as a foundation for the broader printability assessment framework in this work.

To prevent cross-contamination and ensure consistent performance of the MBB system, a rigorous cleaning protocol was implemented. If bioprinting was to be continued without powering off the system, the device was flushed at least three times with bleach, followed by sterile deionized water in between. In cases where the system was shut down between experiments, a five-cycle flushing process was applied. During each cleaning cycle, the valves were held open (manually) using a handheld magnet to allow full perfusion of the cleaning solution. A minimum of 30 s of continuous flow was applied in each step to effectively remove trapped air bubbles and residual bioinks from tubing and internal channels.

### 2.6. Wettability and contact angle measurements

Static contact angle measurements were performed using an Automated Goniometer/Tensiometer (Ramé-hart 260) equipped with DROPimage Advanced software (figure 1(E)). Prior to measurement, the system was calibrated and leveled according to the manufacturer’s protocol to ensure accurate baseline determination. Each bioink was loaded into a micro-syringe and dispensed onto sterile 18 × 18 mm cover glass slides (VWR) under ambient conditions. For each bioink, droplets were deposited at a minimum of three distinct positions on the slide.

Measurements were recorded at 0.1 s intervals, capturing at least 10 time-points per droplet. Contact angle values were calculated using the DROPimage software and averaged to obtain a representative value. For every experimental condition, measurements were performed in triplicate, with independently prepared slides and droplets, to ensure reproducibility and statistical significance.

Between different bioink samples, the dispensing setup used for contact angle measurements was thoroughly flushed with sterile water and dried using compressed air to prevent cross-contamination and ensure consistent droplet deposition.

### 2.7. 3D bioprinting of constructs and cell viability analysis

For each bioink type, 1000 droplets were deposited at a cell density of 0.5 × 10^6^ cells ml^−1^ to fabricate cell-laden constructs as shown in figure 1(E). These droplets were bioprinted on a single plane in a 2D array form, which coalesced into cell-laden bulk constructs. For collagen type I and Matrigel, constructs were thermally gelled at 37 °C for 30 min to induce fibrillogenesis and polymerization, respectively. For alginate, ionic crosslinking was facilitated by gently adding sterile-filtered (via a 0.22 *µ*m filter) calcium chloride (CaCl₂) solution (100 mM), which was prepared under sterile conditions using autoclaved deionized water prior to bioprinting of alginate constructs. CaCl₂ solution covered the alginate constructs for 5 min, followed by a rinse with sterile DPBS (1X) to remove excess ions. For fibrinogen, no thrombin crosslinker was added to the bioprinted constructs, as the goal was to evaluate intrinsic jetting and short-term viability rather than gelation behavior. Growth medium was then added to all samples and constructs were cultured under standard conditions.

Cell viability was assessed at three time points: 3 h, Day 1, and Day 3 post bioprinting using LIVE/DEAD staining (Invitrogen). The staining solution was prepared by mixing 0.15 mM Calcein AM and 2 mM ethidium homodimer-1, which was then added to each construct. Fluorescent images were acquired using a fluorescence microscope (Axio zoom, Zeiss), capturing multiple regions per construct. In addition, cytoskeletal organization was visualized by staining bioprinted NHDFs with Alexa Fluor 568-conjugated phalloidin (Molecular Probes, A12380). Cells were fixed with 4% paraformaldehyde for 2 h at RT, washed with 1×DPBS, and counterstained with Hoechst (Life Technologies) for nuclear visualization. Imaging was performed using the Axio zoom fluorescence microscope. All images were acquired as snapshots without the use of *z*-stacking. To ensure representative visualization in thick constructs, imaging was conducted near the central plane of each droplet, where cells were more evenly distributed, and exposure times were optimized to minimize background. For each construct, average cell viability (%) was calculated from ImageJ (NIH) analysis by dividing the number of live cells (green) by the total number of cells (green+red) and multiplying by 100.

### 2.8. Power-law model and curve fitting

The rheological data were imported to MATLAB Curve Fitting Toolbox (The MathWorks, Inc.) to fit them into a power-law viscosity model [22].

\begin{equation*}\mu = K{\dot \gamma ^{n - 1}}\end{equation*}

where $\mu $ is the apparent viscosity, $K$ is the consistency index, $n$ is the power law index, and $\dot \gamma $ is the deviatoric shear rate of the fluid. We selected nonlinear weighted least square minimization method for parameter optimization. For the choice of weights, we followed the work of Destrade *et al*, where the choice of weights was inverse square of the data points $\left( {{w_i} = \frac{1}{{\mu _i^2}}} \right)$ [23]. Levenberg–Marquardt algorithm was used as the solver. The obtained rheological parameters for the power-law viscosity model were imported to COMSOL Multiphysics® for finite element (FE) simulations [24].

### 2.9. FE simulations

The MBB operates through a valve-actuated mechanism, where the valve opens for a dwell time of 1 ms to eject bioink. To investigate the flow dynamics and shear effects on cells occurring during this brief valve actuation, the cylindrical bioprinter nozzle was modeled using a 2D axisymmetric formulation available in the simulation software. The nozzle geometry was represented as a rectangular domain corresponding to a physical nozzle length of 0.84 mm and an internal radius of 0.125 mm. The fluid behavior was characterized using the built-in power-law rheological model, with parameters obtained from experimental rheological fittings of the bioink. A fully developed inlet flow boundary condition was applied, with the flow rate determined from the ratio of the ejected ligament volume to the valve dwell time. The nozzle wall was assigned a no-slip boundary condition, while the outlet was defined by a zero-gauge pressure condition. The computational domain was discretized using a structured mesh composed of triangular elements, with maximum and minimum element sizes of 8.38 × 10^−4^ and 2.5 × 10^−6^ mm, respectively. The default P1–P1 stabilized element pair was selected for simulation with stationary solver to simulate steady-state solutions.

### 2.10. Statistical analysis

Data analysis and visualization were performed using Prism 8 (GraphPad). Differences among multiple groups were evaluated using one-way analysis of variance followed by Tukey’s post-hoc test. Comparisons between a specific group and its control were conducted using a two-tailed t-test. Statistical significance was set at ^*^P < 0.05, ^**^P < 0.01, and ^***^P < 0.001.

## 3. Results and discussion

### 3.1. Shear-thinning and viscoelastic properties of bioinks

**Figure 2.**
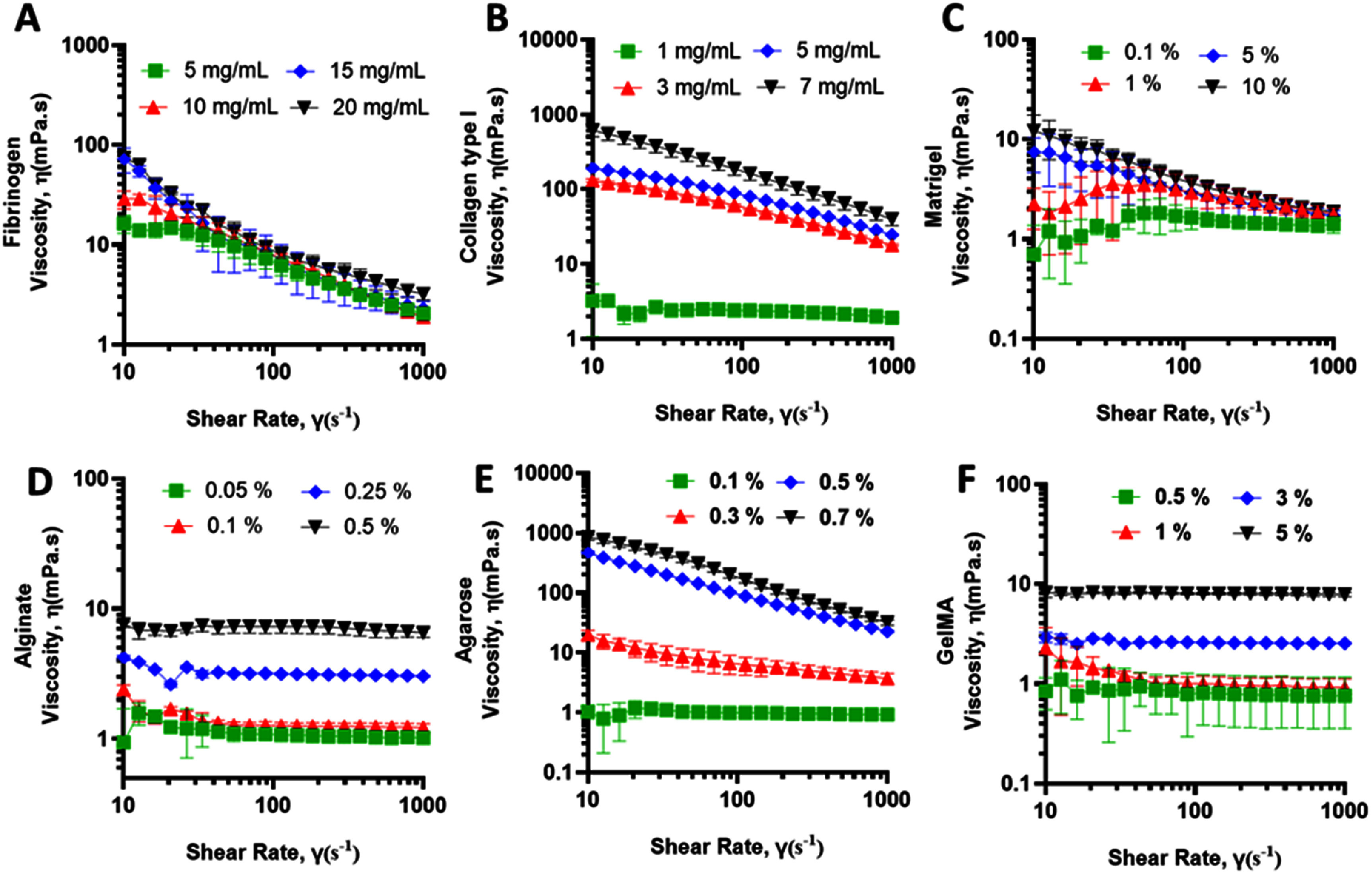
Viscosity (*η*) profiles of bioinks as a function of shear rate for surfactant-free solutions. (A) Fibrinogen, (B) collagen type I, (C) Matrigel, (D) alginate, (E) agarose, and (F) GelMA. Error bars represent SD (*n* = 3).

*η* profiles of the tested bioinks (figure 2), including fibrinogen, collagen type I, Matrigel, alginate, agarose and GelMA, displayed distinct rheological signatures, each directly impacting their performance in MBB. All bioinks exhibited non-Newtonian, shear-thinning behavior, characterized by a decrease in viscosity with increasing shear rate. This shear-thinning response is particularly advantageous for MBB, as the reduced *η* at high shear facilitates droplet ejection when pressure pulses are applied, whereas the higher zero-shear *η* supports droplet integrity and minimizes undesirable spreading or rebound upon substrate impact [25, 26]. The balance between *η* and pressure was critical: more viscous bioinks require higher driving pressures or longer valve-open times for ejection, but excessive pressure increases the risk of satellite droplet formation [4, 5]. Moreover, increasing shear *η* primarily suppresses satellite formation and improves droplet-to-droplet uniformity; droplet size itself was governed by the actuation conditions (pulse width/pressure) and nozzle diameter, rather than *η* per se [27–29]. Collectively, these rheological characteristics highlight *η* as a key determinant of both droplet generation and structural stability in MBB.

Quantitative rheological analysis revealed pronounced differences among the bioinks as a function of concentration (figure 2). Fibrinogen exhibited a noticeable decrease in viscosity with increasing shear rate; however, the overall magnitude of this change was limited across the tested shear-rate range, indicating weak shear-thinning behavior and low shear sensitivity relative to other bioinks evaluated in this study (figure 2(A)). Collagen type I demonstrated the most pronounced shear-thinning response, with viscosity spanning nearly an order of magnitude between low and high shear rates, reflecting its strong fibrillar network formation (figure 2(B)). Matrigel remained comparatively soft and viscous-dominant, with viscosity decreasing gradually and remaining within a narrow range, suggesting limited structural rearrangement under shear (figure 2(C)). Alginate showed intermediate behavior, with viscosity moderately decreasing across the shear rate range and a clear monotonic increase in viscosity with concentration, consistent with progressive chain entanglement and ionic crosslinking effects (figure 2(D)). Agarose exhibited strong concentration-dependent viscosity with pronounced shear-thinning at higher concentrations, indicating the formation of a dense, physically crosslinked network that was highly sensitive to shear deformation (figure 2(E)). GelMA showed relatively low viscosity and weak shear-rate dependence across concentrations, with only modest shear-thinning, reflecting its low degree of physical entanglement prior to photo-crosslinking (figure 2(F)).

To enable a more accurate interpretation, rheological comparisons were discussed within each bioink system rather than across different formulations, since differences in concentration, molecular structure, and polymer network density can confound direct viscosity comparisons.

**Figure 3.**
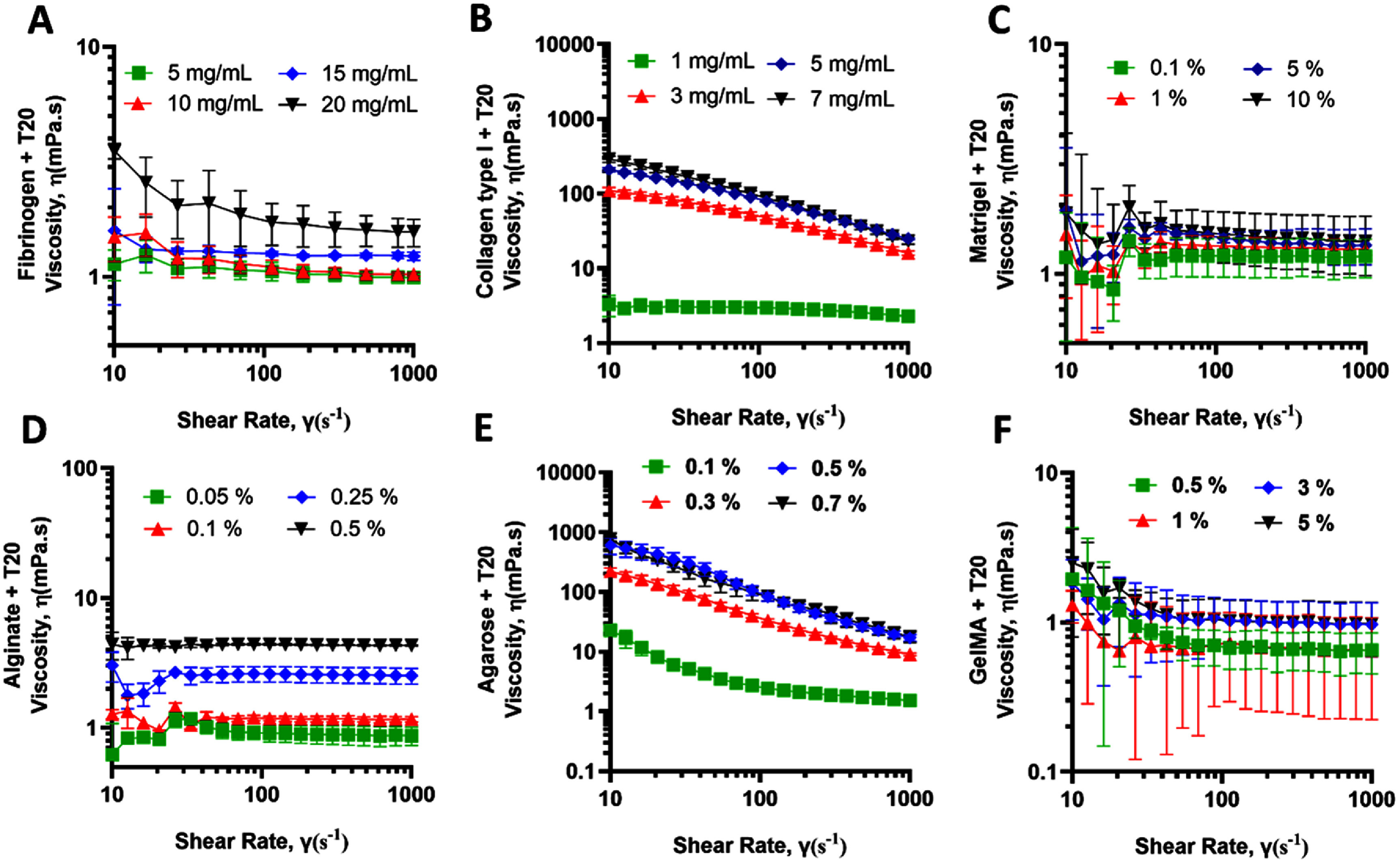
Viscosity (*η*) profiles of bioinks supplemented with T20 as a function of shear rate. (A) Fibrinogen, (B) collagen type I, (C) Matrigel, and (D) alginate, (E) agarose, and (F) GelMA. Error bars represent SD (*n* = 3).

A trace amount of surfactant T20 (⩽0.001% v/v) was included to minimize interfacial artifacts such as edge fracture, surface slip, and meniscus instabilities that commonly occur in non-Newtonian, protein-rich bioinks [30–33]. We selected this low concentration to avoid cytotoxic effects, as prior studies have shown that T20 concentrations below 0.01% maintain greater than 90% cell viability across multiple cell types (e.g. PK-15 and B16-F10), whereas higher levels (⩾0.03%) cause morphological changes and decreased viability [19, 34–36]. This minimal addition improved sample wetting and reproducibility without affecting bulk viscosity or viscoelastic moduli [37–39]. In bioprinting experiments, T20 further fine-tuned interfacial behavior by lowering surface tension and contact angle, thereby shortening capillary pinch-off time, enhancing droplet detachment, reducing nozzle wetting, and suppressing satellite formation [40–42]. Consistent with these observations, each bioink retained its characteristic shear-thinning response, exhibiting a continuous decrease in viscosity with increasing shear rate. Addition of T20 did not substantially alter the bulk flow behavior of the bioinks (figure 3). Across all concentrations, viscosity profiles showed comparable trends to the surfactant-free controls, with deviations generally within ±10%–15% which are within the expected experimental variability for protein-based bioinks.

Fibrinogen exhibited a moderate decline in viscosity with increasing shear rates; however, the addition of T20 led to a marked reduction in viscosity, particularly at higher concentrations, suggesting that the surfactant disrupted weak intermolecular associations within the fibrinogen network (figure 3(A)). Collagen type I remained the most shear-sensitive material. Although T20 slightly reduced its viscosity at low shear rates (⩽15%), the overall trend was unchanged (figure 3(B)). Matrigel continued to display low viscosity and limited shear dependence, consistent with its soft, weakly structured matrix, where T20 induced negligible variation (figure 3(C)). Alginate retained its intermediate response, showing small viscosity reductions at low shear while preserving the overall curve shape (figure 3(D)). Agarose exhibited pronounced shear-thinning even in the presence of T20, with strong concentration-dependent viscosity maintained across the full shear-rate range, indicating that its physically-crosslinked network was not disrupted by surfactant addition (figure 3(E)). GelMA showed low viscosity and weak shear-rate dependence across concentrations, with T20 inducing only minor reductions in low-shear viscosity and no qualitative change in flow behavior, consistent with its low pre-crosslinking entanglement density (figure 3(F)).

### 3.2. Concentration-driven changes in bioink storage and loss modulus

Rheological properties of bioinks are critical determinants of their printability, droplet formation dynamics, and the stability of resulting constructs. Among these properties, *G′* and *G″* provide direct insight into the relative contributions of elastic (solid-like) and viscous (liquid-like) behavior, respectively. Modulating bioink concentration is one of the most straightforward yet powerful strategies to tune viscoelasticity, as it directly influences polymer chain entanglement, fibrillar network density, and intermolecular interactions. Numerous studies have demonstrated that increasing bioink concentration consistently elevates both *G′* and *G″*, although the extent of this increase and the balance between elasticity and *η* remain strongly dependent on the bioink’s intrinsic molecular architecture and crosslinking potential [43–46].

**Figure 4.**
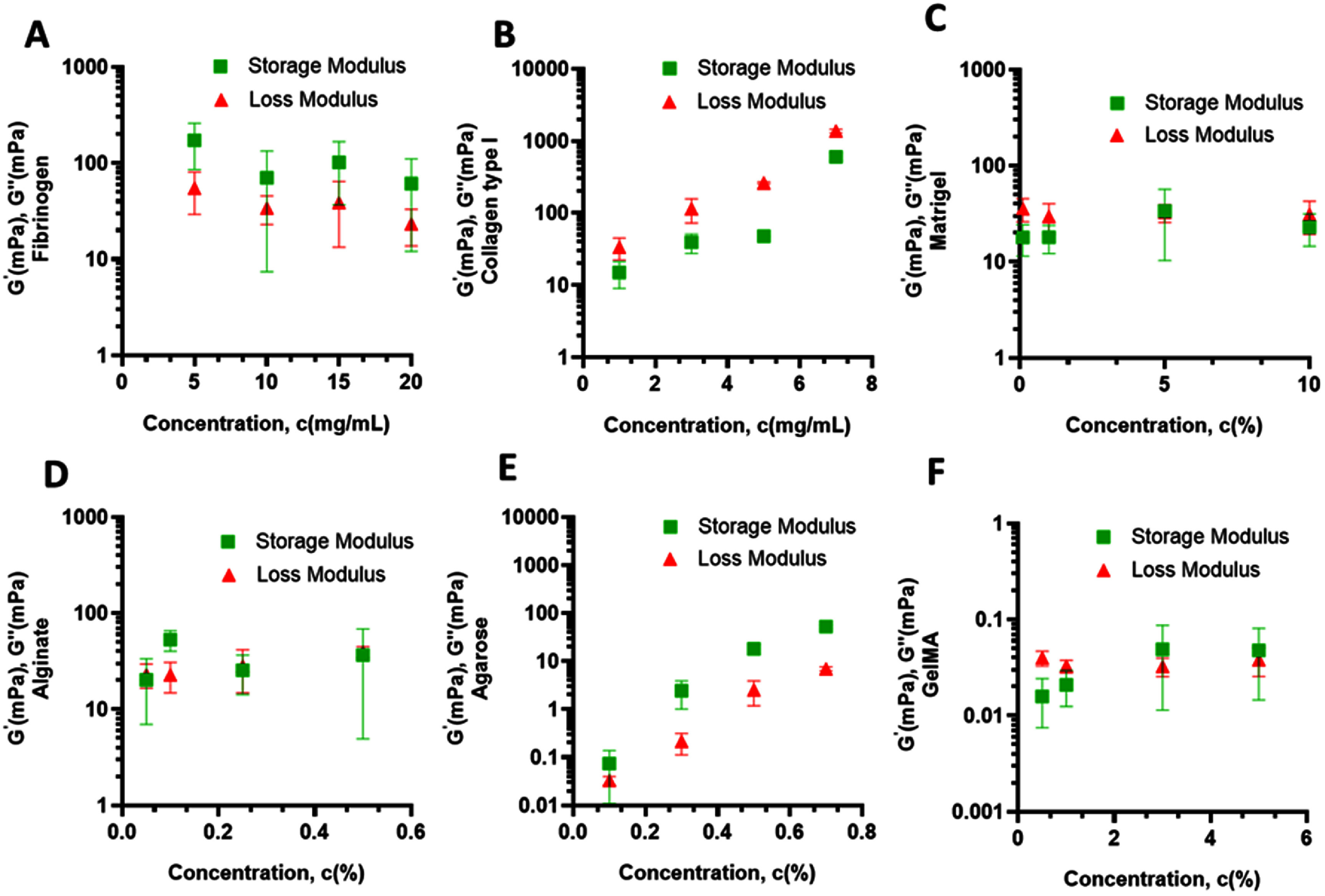
Storage (G′) and loss modulus (*G″*) of surfactant-free bioinks at different concentrations. (A) Fibrinogen, (B) collagen type I, (C) Matrigel and (D) alginate, (E) agarose, and (F) GelMA. Error bars represent SD (*n* = 3).

These concentration-dependent rheological shifts directly affect fluid breakup mechanics in DBB. As viscosity and elasticity increase, the fluid’s resistance to deformation and extensional thinning becomes greater, influencing the balance between inertial, viscous, capillary, and gravitational forces that govern jetting behavior. These effects are quantitatively captured by the Weber (*We*), Reynolds (*Re*), Ohnesorge (*Oh*), and Froude (*Fr*) numbers, which together define the operating regime of a given bioink. Specifically, increasing viscosity or elasticity raises *Oh* and lowers *Re*, shifting the system toward viscosity-dominated flow; decreasing surface tension increases *We* and accelerates pinch-off; and increasing density or actuation velocity modifies *Fr*, influencing the relative contribution of gravity to droplet detachment [47, 48]. In the following sections, these relationships are analyzed through the dimensionless framework to evaluate how concentration-dependent rheological changes in *G′* and *G″* translate into distinct printability regimes for each bioink (figure 4).

Fibrinogen showed only a modest increase, with both moduli rising from ∼5–20 mPa at 5 mg ml^−1^ to ∼30–100 mPa at 20 mg ml^−1^ (figure 4(A)). Here, *G′* and *G″* remained closely coupled, suggesting that fibrinogen solutions retained a predominantly viscous character in the absence of thrombin-driven crosslinking. Such modest moduli changes maintain low *Oh* and relatively high *Re*, yielding energetic breakup and greater satellite risk or *μ* is increased within biocompatible limits so that *Z* enters the printable band. In contrast, collagen type I exhibited the steepest concentration-driven stiffening, with *G′* and *G″* rising 10–30 mPa range at 1 mg ml^−1^ to ∼100–300 mPa at 7 mg ml^−1^ (figure 4(B)). Importantly, *G′* increased more rapidly than *G″*, indicating a transition toward elastic-dominant behavior as fibrillar networks densified through triple-helix entanglement. Rapid *G′* growth at high concentration adds elastic resistance to filament thinning, reducing ejected volume per pulse and shortening the ligament at the same dwell and pressure. Clean pinch off is recovered, either by raising the *We* number or by lowering *μ* when high *G′* is not required for shape fidelity. Excessive elasticity shifts the system toward high *Oh* and low *Re*, suppressing satellite formation but increasing the risk of incomplete detachment unless *We* is increased by higher velocity or lower *σ*.

Matrigel maintained uniformly low values across concentrations, with *G′* and *G″* generally below 20–40 mPa even at 10% (figure 4(C)). Low *G′* and *G″* maintain a viscous dominant response, and droplets spread and coalesce more readily. Moreover, *G″* was consistently comparable to or slightly higher than *G*′, reflecting weak elastic reinforcement and a viscous-dominant response characteristic of its loosely organized basement membrane proteins. Before crosslinking, moderate increases in *G′* and *G″* keep *Oh* in a mid-range that supports stable thinning with low satellite propensity and predictable dot size while higher concentration improves post-deposition shape retention. By comparison, alginate demonstrated a clear concentration-dependent rise, with moduli increasing from ∼10–30 mPa at 0.05%–0.1% to ∼50–100 mPa at 0.5% (figure 4(D)). Like collagen type I, *G′* grew more steeply than *G″*, pointing to enhanced elasticity through polysaccharide chain entanglement, which shifted its behavior toward a solid-like regime at higher concentrations. Agarose exhibited a pronounced concentration-dependent stiffening, with both *G′* and *G″* increasing by nearly two orders of magnitude from 0.1% to 0.7% (figure 4(E)). At higher concentrations, *G′* strongly exceeded *G″*, indicating a transition to an elastic-dominant, physically crosslinked network that substantially increases *Oh* and suppresses extensional thinning, thereby shortening ligament length and narrowing the printable window. GelMA showed the lowest moduli among all bioinks, with *G′* and *G″* remaining in the 0.01–0.1 mPa range even at 5% (figure 4(F)). The weak viscoelastic response prior to photo-crosslinking maintains very low *Oh* and high *Re*, favoring rapid thinning and clean detachment but increasing sensitivity to satellite formation unless surface tension and actuation velocity are carefully controlled.

At high concentrations, collagen’s bulk viscoelastic behavior was dominated by its *G′* and *G″*, thereby increasing *We* and shortening the capillary pinch-off time. Adding T20 lowers *σ* without materially changing *G′* or *G″*, which raises *We* and shortens the capillary pinch-off time. Practically, this helps thick collagen jets thin and detach at the same actuation settings, reducing incomplete detachment events. Alginate already detaches robustly in our baseline regime. With T20, the lower *σ* preserves clean detachment without increasing satellite droplets. Fibrinogen operates at high *We* and relatively low *Oh*. Lowering *σ* with T20 pushes the operating point toward the satellite-forming region. To avoid satellites, a slight reduction in jet velocity is required. For Matrigel, lower *σ* with unchanged bulk moduli can enhance spreading, so dot size calibration should be rechecked. In practical terms, concentration tunes *μ* and *G′* and therefore *Oh* and *Re*, whereas T20 tunes *σ* and therefore *We*, and their balance governs ligament length, pinch off, satellite propensity, and final dot size in MBB.

### 3.3. Ligament dynamics and droplet formation

#### 3.3.1. Droplet diameter analysis

Droplet diameter is a key determinant in MBB because it directly affects printing resolution, deposition fidelity, and the structural integrity of constructs [5, 49]. The diameter of droplets results from the interplay between inertial, capillary, and viscous forces, all of which are strongly modulated by the rheological properties of a bioink [4, 50, 51]. Among these properties, polymer or protein concentration plays a central role, with higher concentrations enhancing *η* and viscoelasticity, causing higher resistance to droplet deformation and restricting it from spreading and providing enhanced control over breakup during ejection. It also favors the formation of smaller, more uniform droplets because higher *η* dampens instabilities and inhibits the formation of undesirable satellite droplets [52–55]. At lower concentrations, reduced chain entanglement or weaker network formation allows the generation of larger, more variable droplets, often compromising printing stability. Understanding how different classes of bioinks respond to concentration changes is therefore critical, as protein-rich bioinks, such as collagen, fibrinogen, and Matrigel, typically show pronounced sensitivity, whereas polysaccharide based systems, such as alginate, exhibit more uniform droplet behavior [31, 56, 57]. This knowledge provides valuable guidance for selecting and tuning bioinks to achieve reproducible and high-fidelity bioprinting outcomes in MBB.

**Figure 5.**
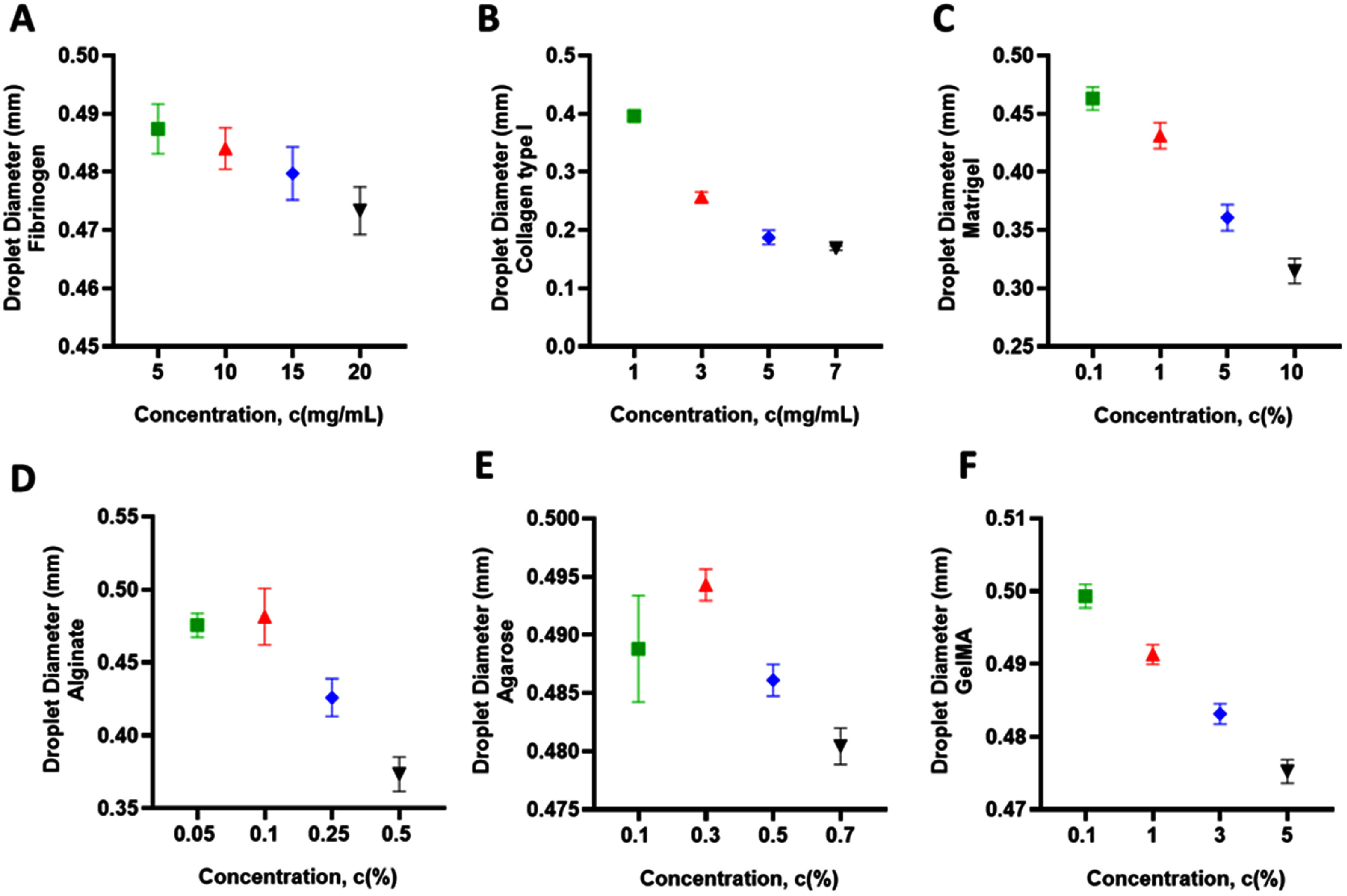
Droplet diameter across the six bioinks and concentrations (A) Fibrinogen, (B) collagen type I, (C) Matrigel, (D) alginate, (E) agarose, and (F) GelMA. Error bars represent SD (*n* = 3).

Droplet diameter measurements revealed clear concentration-dependent effects across all bioinks (figure 5). For fibrinogen, increasing concentration from 5 to 20 mg ml^−1^ led to a gradual reduction in droplet diameter, reflecting enhanced *η* and reduced spreading with higher protein content (figure 5(A)). Collagen type I exhibited a marked decrease in droplet diameter as concentration increased from 1 to 7 mg ml^−1^, consistent with its steep rise in viscoelasticity at higher concentrations (figure 5(B)). Matrigel demonstrated a comparable trend, with droplet diameter decreasing progressively from 0.1% to 10%, indicating strong sensitivity of droplet size to gel concentration (figure 5(C)). In contrast, alginate displayed relatively moderate reductions in droplet diameter from 0.05% to 0.5%, suggesting that its droplet formation dynamics were less influenced by concentration changes within the tested range compared to protein-rich bioinks (figure 5(D)). Agarose showed a clear concentration-dependent decrease in droplet diameter from 0.1% to 0.7%, reflecting the rapid stiffening of its physically crosslinked network and the associated shortening of ligament length at higher concentrations (figure 5(E)). GelMA likewise exhibited a systematic decrease in droplet diameter from 0.5% to 5%, despite its low absolute modulus, indicating that even weak increases in pre-crosslinking viscoelasticity are sufficient to reduce ejected volume and final droplet size (figure 5(F)). Across all bioinks, adding T20 at 0.001% v/v did not produce a significant change in mean droplet diameter under fixed dwell and back pressure while the primary effect was reduced satellite formation and improved droplet uniformity.

When comparing bioinks, collagen type I and agarose produced smaller droplets at equivalent concentrations relative to alginate and Matrigel, highlighting the stronger impact of protein network formation on droplet pinch-off and final size. Matrigel and GelMA exhibited the largest droplets at low concentrations, their weak pre-crosslinking network structure and higher intrinsic *η*. Alginate droplets remained more uniform across concentrations, underscoring its relatively stable droplet formation behavior compared to protein-based bioinks. Collectively, these findings demonstrate that protein-rich inks (collagen type I, fibrinogen, Matrigel) are more sensitive to concentration changes, whereas alginate offers more consistent droplet diameters, a property favorable for reproducible MBB. The addition of T20 did not alter these relative rankings, but it improved dispensing consistency by suppressing satellites and slightly narrowing the coefficient of variation of droplet size, without a measurable shift in the mean diameter at fixed actuation.

#### 3.3.2. Ligament volume analysis

In MBB, the ligament is the transient fluid column connecting the emerging droplet to the nozzle immediately before the pinch-off [10, 28, 58]. Its volume is determined by the balance of inertial, viscous, elastic, and capillary forces during extensional thinning, making it a sensitive readout of bioink formulation and cell loading [51, 59, 60]. Larger ligaments reflect greater spreading potential and slower capillary drainage, whereas smaller ligaments indicate faster pinch-off and more confined deposition [50, 61]. Because ligament volume is established prior to droplet contact with the substrate, it provides a practical handle for tuning print fidelity upstream in the bioprinting process [40, 62, 63].

**Figure 6.**
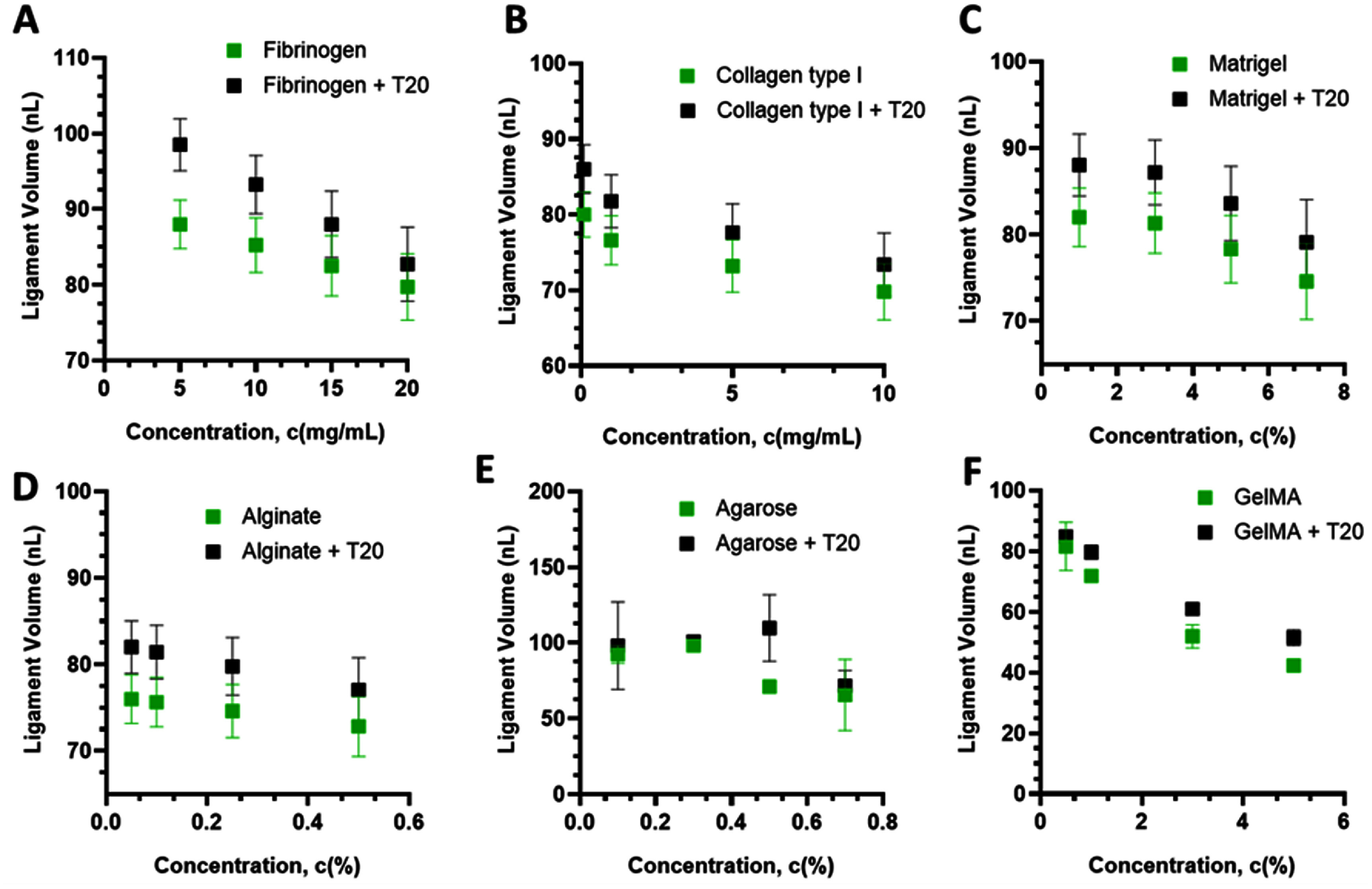
Ligament volume versus bioink concentration with and without T20 for (A) fibrinogen, (B) collagen type I, (C) Matrigel, and (D) alginate, (E) agarose, and (F) GelMA. Error bars represent SD (*n* = 3).

At fixed actuation with dwell and back pressure held constant, the ligament volume decreased as concentration increased for all bioinks (figure 6). This behavior consists of higher *η* and weak elasticity raising *Oh* and lowering *Re*, which reduces the expelled volume for the same impulse. Fibrinogen exhibited a modest monotonic decrease in ligament volume with an increase in concentration from 5 to 20 mg ml^−1^, reflecting low *Oh* (figure 6(A)). The largest relative drop was observed for collagen type I, where ligament volume decreased from ∼82–85 nl at 1 mg ml^−1^ to ∼70–75 nl at 7 mg ml^−1^, corresponding to a reduction of ∼10%–15% (figure 6(B)). The most pronounced decrease occurred between 1 and 3 mg ml^−1^, after which the values plateaued. Matrigel showed an intermediate sensitivity with a stepwise decline and low variability that indicates a modest increase in flow resistance without a strong elastic contribution (figure 6(C)). Alginate displayed the smallest concentration sensitivity over 0.05%–0.5% with only a gradual reduction in volume and low dispersion, consistent with a viscous dominant solution whose *Oh* increases only mildly in this range (figure 6(D)). Agarose exhibited a clear concentration-dependent decrease in ligament volume from 0.1% to 0.7%, with a pronounced drop between 0.3% and 0.5%, reflecting the rapid stiffening of its physically crosslinked network and the associated increase in *Oh* that shortens the ejected ligament (figure 6(E)). GelMA showed a systematic and relatively large decrease in ligament volume from 0.5% to 5%, indicating that even weak increases in pre-crosslinking viscoelasticity substantially reduce the expelled volume under fixed actuation (figure 6(F)).

Introducing T20 across the same concentration series did not produce a systematic shift in the mean ligament volume for any of the bioinks. T20 curves overlapped the surfactant-free data within experimental variability (typically ⩽5%–10%). The most consistent effect of T20 was a slight tightening of the dispersion (smaller SD), in line with the expectation that lowering *σ* improves pinch-off regularity without significantly altering bulk ejected volume under small-strain, valve-driven actuation. With actuation parameters held constant, variations in ligament volume reflected changes in *η* (and weakly elasticity) with concentration; T20 chiefly tightened run-to-run variability without shifting the mean.

To evaluate whether the presence and density of suspended cells influence jetting dynamics and ligament formation, we next examined cell-laden bioinks. In practice, encapsulated cells can slightly alter local viscosity, elasticity, and interfacial behavior due to cell-matrix interactions [64–66]. To isolate the effect of cell loading itself, each bioink was tested at its lowest bioink concentration, minimizing bioink-induced viscosity effects while maintaining printability. This approach enabled a direct assessment of how increasing cell density influences droplet ejection stability and ligament volume consistency, providing insight into the transition from acellular to biologically relevant printing conditions.

**Figure 7.**
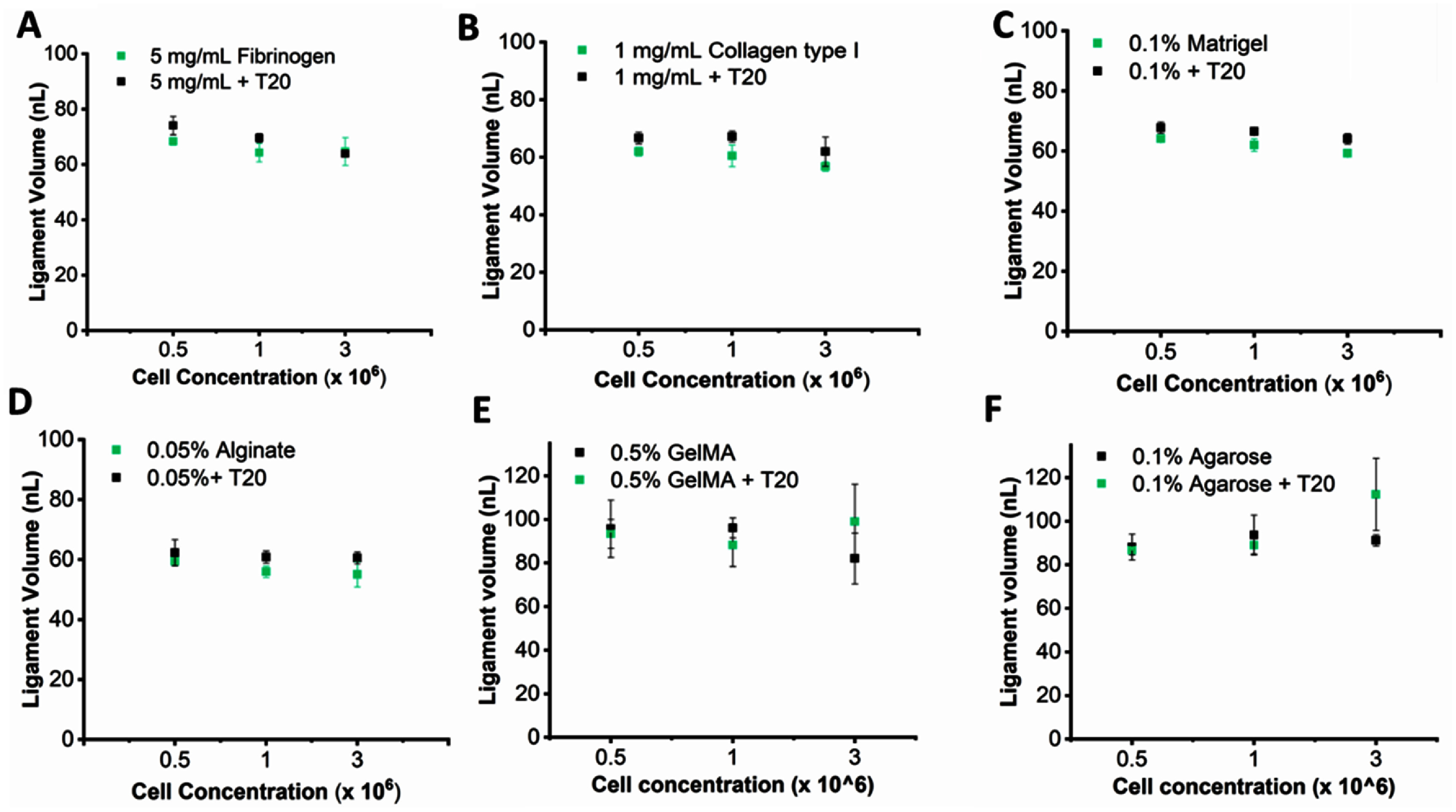
Ligament volume versus cell concentration at a fixed bioink concentration. For each bioink (at its lowest concentration), ligament volume was measured at 0.5, 1, and 3 × 10^6^ cells ml^−1^ with and without T20 including (A) fibrinogen, (B) collagen type I, (C) Matrigel, and (D) alginate, (E) agarose, and (F) GelMA. Error bars represent SD (*n* = 3).

With bioink concentrations fixed at their minimum and cell loading density varied from 0.5 to 3 × 10^6^cells ml^−1^, ligament volumes for all bioinks remained within a narrow (55–75 nl) band (figure 7). At 0.5 × 10^6^ cells ml^−1^, volumes for fibrinogen, collagen type I, Matrigel, and alginate were statistically indistinguishable. Increasing to 1 × 10^6^ cells ml^−1^ produced changes of 1–10 nl with no consistent upward or downward shift across bioinks, indicating that the modest increase in effective *η* at this volume fraction did not significantly alter *Oh* or *Re* at the operating velocity. At 3 × 10^6^ cells ml^−1^, collagen type I showed a small downward shift relative to its lower cell loading density, consistent with greater sensitivity to extensional resistance, whereas fibrinogen, Matrigel, and alginate indicated no significant reduction in ejected volume across this practical range of cell densities (figures 7(A)–(D)). GelMA likewise showed no monotonic decrease in ligament volume with increasing cell concentration, with values remaining within ±10 nl across the tested range, indicating that cell loading did not substantially perturb its low pre-crosslinking viscoelasticity (figure 7(E)). Agarose exhibited similarly weak sensitivity to the cell concentration, with ligament volumes remaining nearly constant from 0.5 to 3 × 10^6^ cells ml^−1^ despite its higher baseline stiffness, demonstrating that cell loading did not significantly modify ejected volume in physically crosslinked networks at these concentrations (figure 7(F)).

Introducing T20 at 0.001% did not produce a systematic change in mean ligament volume at any cell concentration for any of the bioinks; it overlapped with the surfactant free data within experimental variability. This outcome is consistent with T20 mainly lowering *σ* and increasing *We*, which suppresses satellites and tightens droplet to droplet variability, while the bulk ejected volume remains governed by actuation and matrix concentration rather than by cell loading in the tested range.

#### 3.3.3. Analysis of dimensionless numbers

In DBB, fluid breakup behavior is governed by the interplay of inertial, viscous, capillary, and gravitational forces. Quantifying these forces through dimensionless numbers, such as *We, Re, Oh*, and *Fr*, provides a universal framework to compare bioink performance across different formulations and concentrations [4, 50, 67, 68] The detailed definitions and calculation methods for these dimensional numbers are provided in the supplementary material. These parameters are particularly valuable in MBB, where subtle variations in bioink rheology or *σ* can determine whether droplets detach cleanly, form elongated ligaments or generate satellites. These numbers provide a formulation-agnostic way to compare bioinks and to anticipate whether ejection will yield clean droplets, long-lived filaments, or satellites. In particular, low *Oh* (equivalently high *Z* = 1/*Oh*) identifies an inertio-capillary regime where *η* is weak and breakup is governed by inertia and *σ*. A broad body of literature in the context of inkjet printing places robust single-drop operation within *Z* of about 1–10 (i.e., *Oh* of about 1 to 0.1), with satellites common when *Z* ≳ 10 and viscous or energy-limited behavior when *Z* ≲ 1 [69–72]. Throughout, adding T20 reduces *σ* and therefore increases both *We* and *Oh* at fixed *D* (nozzle orifice diameter), shifting points toward higher jetting energy but also stronger viscous damping.

For fibrinogen samples, across 5–20 mg ml^−1^, fibrinogen remained squarely inertio-capillary: *We* changed from 105.39–95.97, *Re* from 1191.5–773.0, *Oh* from 0.0086 to 0.0127, and *Fr* from 96.278 to 88.803. Low *Oh* and high *Re* explain the rapid thinning and the satellite propensity when the impulse is large (table S1). T20 reduced *σ* substantially and therefore increased *We* (from 95.968 to 107.96 over the same concentration range) and slightly increased *Oh* (from 0.0127 to 0.0134). Consistent with this, ligament aspect ratio rose (e.g. ${L_{{\mathrm{ar}}}}$ from 3.630 to 4.098 at 5 mg ml^−1^), improving detachment but demanding careful moderation of dwell or frequency to suppress satellites which is an expected outcome for high-*Z* fluids in inkjet printing.

For collagen type I, all concentrations produced the largest migration across regimes (table S2). At 1 mg ml^−1^, we measured *We* = 23.813, *Re* = 95.011, *Oh* = 0.051, *Fr* = 62.389: corresponding to a low *Oh* inertio-capillary regime in which droplet breakup occurs rapidly and produces compact, well-defined droplets. At 7 mg ml^−1^, *We* fell to 0.895, *Re* to 0.163, *Oh* rose to 5.806, and *Fr* to 26.713, placing collagen type I in a *η*-dominated, energy-limited regime where necking progresses slowly and the ligament remains elongated for a longer duration before detachment. T20 shifted points to higher *We* and higher *Oh* (for example, at 1 mg ml^−1^: *We* = 38.061, *Oh* = 0.064; at 7 Mg ml^−1^: *We* = 1.303, *Oh* = 6.738). Experimentally, ligament length (${L_{\mathrm{l}}})$ and aspect ratio (${L_{{\mathrm{ar}}}})$ increased modestly with T20 (e.g., ${L_{{\mathrm{ar}}}}$ from 1.725 to 2.181 at 1 mg ml^−1^; from 0.335 to 0.404 at 7 mg ml^−1^), indicating easier detachment at low concentrations and slightly longer filaments at high concentrations; however, concentrated collagen still requires either *η* reduction or increased jet velocity to achieve clean pinch-off.

At lower Matrigel concentrations (0.1%–1%), droplet formation was governed mainly by inertia and *σ*, whereas at higher concentrations (5%–10%), *η* played the dominant role in ligament breakup and droplet dynamics (table S3). From 0.1% to 10%, *We* decreased from 65.573 to 9.310, *Re* decreased from 637.390–14.836, *Oh* increased from 0.0127 to 0.2057, and *Fr* decreased from 86.199 to 48.116. As Matrigel concentration increased (from 5 to 10%), *We* fell (from 16.25 to 9.31) while *Oh* rose (from 0.096 to 0.206), consistent with a more viscous, capillary-dominated regime that produces shorter, more damped ligaments and delayed pinch-off. Adding T20 at 10% lowered *σ*, which raised *We* and increased *Oh* (*We* from 9.31 to 14.46 and *Oh* from 0.206 to 0.248). Correspondingly, the measured ${L_{{\mathrm{ar}}}}$ increased from 1.079 to 1.344 and ${L_{\mathrm{l}}}$ grew from 0.270 to 0.336 mm, indicating partially restored detachment under the same actuation. Importantly, these shifts remained within our printable window (stable primary droplets with minimal satellites), which in practice favors mid-range actuation frequencies.

Alginate showed the most concentration-tolerant behavior (table S4). From 0.05% to 0.5%, *We* decreased from 65.708 to 16.452, *Re* decreased from 871.11–57.812, and *Oh* rose from 0.0093 to 0.0702 and *Fr* decreased from 91.147 to 58.347. The entire set stays in or near the printable corridor, with shortening in ligaments with an increase in concentration. T20 increased *We* at each level (from 16.450 to 29.600) and raised *Oh* to 0.0915; ${L_{{\mathrm{ar}}}}$ increased accordingly (from 1.434 to 1.924), consistent with slightly longer but still well-controlled filaments.

For agarose, increasing concentration shifted the system from an inertio-capillary regime to a strongly *η*-dominated regime (table S5). At 0.1%, low *Oh* and high *Re* (*Oh* = 0.0149, *Re* = 510) supported capillary-driven thinning and relatively long ligaments (${L_{{\mathrm{ar}}}}$ = 0.269). At 0.7%, *Oh* increased to 0.905 and *Re* dropped to 8.09, placing the bioink in an energy-limited regime with very short ligaments and strongly damped breakup (${L_{{\mathrm{ar}}}}$ = 0.0044). The addition of T20 increased *We* and *Oh* at all concentrations (e.g., at 0.1%: *We* from 57.46 to 76.62; *Oh* from 0.0149 to 0.0172), slightly easing detachment at low concentrations but not alleviating the strong viscosity limitation at high agarose contents.

For GelMA, increasing concentration from 0.5%–5% drove a transition from inertio-capillary to viscosity-dominated breakup (table S6). At 0.5%, moderate *Oh* and high *Re* (*Oh* = 0.0315, *Re* = 254.3) supported rapid thinning and longer ligaments (${L_{{\mathrm{ar}}}}$ = 0.127), whereas at 5% *Oh* increased to 0.669 and *Re* decreased to 11.97, yielding strongly damped necking and very short ligaments (${L_{{\mathrm{ar}}}}$= 0.006). The addition of T20 increased *We* at all concentrations (e.g. from ∼64 to ∼85) and slightly increased *Oh*, modestly improving detachment at low concentration but leaving high-concentration GelMA in an *η*-limited regime.

The splash parameter (*K*) provides an impact-oriented metric that predicts whether a droplet spreads or splashes upon hitting the substrate [73–75]. High *K* values correspond to greater spreading and potential splashes, while lower *K* values indicate more contained deposition. Collagen type I showed the sharpest decline, with *K* falling from ∼15 at 1 mg ml^−1^ to ∼0.6 at 7 mg ml^−1^, consistent with a transition from inertio-capillary breakup toward viscous-limited behavior that yields compact dots. The addition of T20 raised *K* at a low concentration (to ∼19 at 1 mg ml^−1^), improving detachment, but had negligible effect at a high concentration (remaining <1). Fibrinogen maintained consistently high *K* values (∼60 at 5 mg ml^−1^ to ∼52 at 20 mg ml^−1^), and T20 increased them further (∼55–68), reflecting its tendency to spread readily on impact and underscoring the need to moderate impulse to avoid satellites. For Matrigel, *K* decreased smoothly with concentration (∼41 at 0.1% to ∼6 at 10%), indicating reduced spreading at high solids. T20 partially restored *K* (∼7–8 at 10%), allowing cleaner detachment while keeping the deposition stable. Alginate was the most tolerant system with *K* values decreasing from ∼44 at 0.05% to ∼11 at 0.5%. T20 raised them to ∼15–58 across the same range, but all conditions remained within a controllable window. Agarose exhibited a strong concentration-dependent splashing, with *K* decreasing by more than an order of magnitude from dilute to concentrated formulations, consistent with its rapid transition into a highly viscous, energy-limited regime at high polymer contents. T20 increased *K* modestly at low concentrations, improving detachment, but did not shift concentrated agarose out of the low-*K*, non-splashing regime. GelMA showed an intermediate response, with *K* decreasing monotonically with concentration as viscosity and elasticity increased. The addition of T20 raised *K* at low concentrations, enhancing detachment, while at high concentrations *K* remained low, indicating that impact dynamics were dominated by viscous damping rather than interfacial effects.

Overall, our data show that collagen type I migrates from inertio-capillary to *η*-limited as concentration increases, which explains its need for lower *μ* at the upper end. Fibrinogen stays in the inertio-capillary regime, exhibiting the highest *Re* among and consistently high *We* among all bioinks. This combination facilitates rapid jet formation and droplet ejection, but the elevated inertial energy also increases the likelihood of satellite droplet formation if the actuation impulse is not sufficiently dampened. Matrigel transitions smoothly, where dilute samples are printable and impact-stable, whereas concentrated samples approach energy-limited behavior. T20 helps by lifting *We* without pushing *Re* to extremes. Alginate operated within a wide printable window across its concentration range, maintaining stable primary droplets with low satellite propensity and requiring only minor adjustments to dwell or back-pressure. Agarose displays a pronounced concentration-dependent shift from inertio-capillary to strongly viscosity-dominated behavior, with low concentrations yielding long-lived ligaments and high concentrations producing damped breakup and minimal splashing; surfactant addition modestly improves detachment at low concentrations but cannot overcome viscous suppression at high polymer contents. GelMA demonstrates intermediate behavior between the protein- and polysaccharide-based systems. At low concentrations, moderate viscosity allows clean jetting within the inertio-capillary regime, whereas at higher concentrations, elevated viscosity and viscoelasticity suppress ligament thinning, driving the system toward a non-splashing, energy-limited regime. These trends align with established printability maps in which *Z* between 1 and 10 is broadly printable, *Z* much larger than 10 favors satellites, and *Z* below 1 is too viscous without additional impulse. Taken together with *K*, it complements the *We-Re-Oh* framework by capturing impact outcomes: high *K* bioinks require tighter control to avoid splash and spreading, while lower *K* bioinks favor contained dots and higher printing fidelity.

#### 3.3.4. Printability map

**Figure 8.**
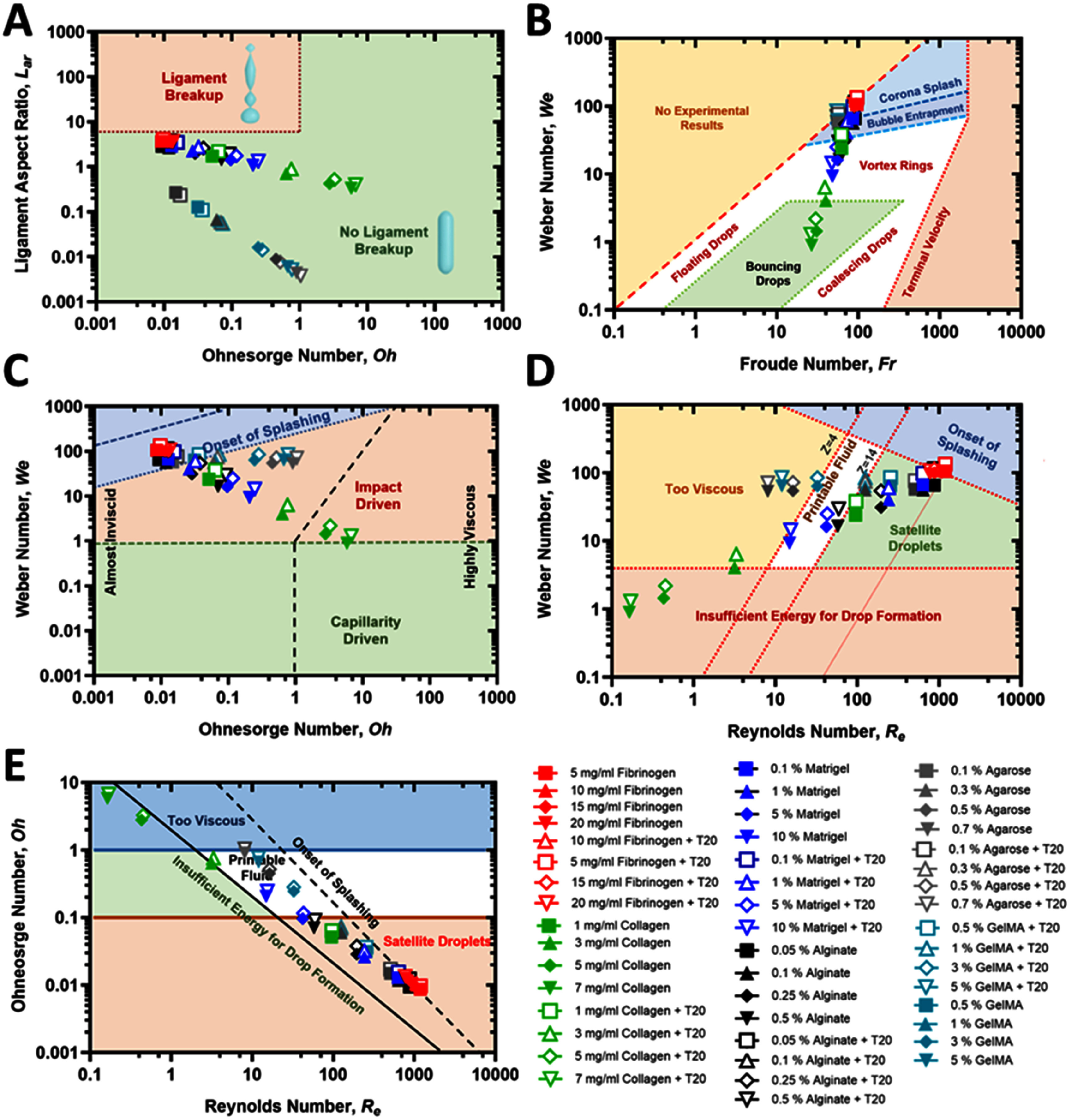
(A) The ${L_{{\mathrm{ar}}}}$ vs *Oh* number map. Shaded regions indicate expected regimes (‘No ligament breakup’ vs ‘Ligament breakup’). (B) The *We*–*Fr* map. Shaded regions summarize canonical impact outcomes (floating/bouncing drops, crown/corona splash onset, and vortex rings). (C) The *We–Oh* printability regime. The diagram delineates thin-liquid/aerodynamically influenced, capillarity-driven, and impact-driven regions, including an ‘onset of splashing’ boundary. (D) The *We*–*Re* map. Relationship between *We* and *Re* highlighting practical boundaries for MBB (too viscous/insufficient energy for drop formation vs. satellite-droplet regime). (E) The *Oh–Re* printability map delineating practical bioprinting regimes for a 250 *μ*m nozzle: ‘Too viscous/insufficient energy,’ ‘Printable fluid,’ and ‘Satellite droplets.’ Each marker pair corresponds to the same formulation with/without T20.

Printability maps provide a powerful framework for interpreting droplet jetting and breakup dynamics in DBB (figure 8). By plotting combinations of dimensionless groups, such as *Oh, We, Re*, and *Fr*, it is possible to delineate distinct physical regimes and identify practical windows for stable droplet formation. Each axis represents a balance of fundamental forces: *We* quantifies inertia relative to *σ* or capillary forces, *Re* compares inertia to viscous dissipation, *Oh* integrates *η* with respect to inertia and capillarity, and *Fr* captures the competition between inertial and gravitational effects [27, 29, 76]. Together, these numbers establish whether a jet is inertia-driven, *η*-damped, capillarity-controlled, or prone to gravitational distortion.

Different regions of these maps correspond to specific droplet outcomes. At low *Oh* and high *Re/We*, jets are inertia-dominated and prone to satellite droplet formation due to rapid ligament thinning [27]. Conversely, high *Oh* and low *Re* characterize viscous- or energy-limited conditions, where droplet detachment is suppressed and long-lived filaments emerge [27]. The intermediate band represents the printable window, where inertial, viscous, and capillary forces are balanced, enabling clean droplet pinch-off without satellites. ${L_{ar}}\,$ further enriches these maps by indicating whether ligaments are short and quickly damped or elongated and susceptible to breakup. These boundaries have been validated across multiple experimental and numerical studies [29, 62, 77].

Mapping ${L_{{\mathrm{ar}}}}$ against *Oh* separates inertio-capillary thinning from *η*-dominated damping, which is directly relevant to how long a filament persists before the pinch-off (figure 8(A)). Across all bioinks, increasing concentration moved points to higher *Oh* and simultaneously lowered ${L_{{\mathrm{ar}}}}$, showing that added solids shorten and damp ligaments. Fibrinogen occupied the lowest *Oh* band, from 0.0086 to 0.0127, with the largest ${L_{{\mathrm{ar}}}}$, from 1.7 to 3.6, consistent with energetic thinning and a higher satellite risk when the drive impulse is large. Collagen type I spanned the highest *Oh* values, from 0.051 at 1 mg ml^−1^ to above 1 at 5–7 mg ml^−1^, with ${L_{{\mathrm{ar}}}}$ from 0.6 to 1.7, indicating *η*-limited necking and a risk of incomplete detachment unless the impulse is increased. Matrigel formed an intermediate band (*Oh* from 0.0127 to 0.206; ${L_{ar}}\,$ from 1.1 to 2.9), while alginate covered low-to-moderate *Oh* (*Oh* from 0.0093 to 0.070; ${L_{ar}}\,$ from 1.4 to 2.9). Agarose occupied the highest *Oh* range among all bioinks (*Oh* from 0.051 to 5.81 without T20 and up to ∼6.74 with T20), with ${L_{ar}}\,$ consistently below ∼1, indicating strongly damped, short ligaments and a narrow printable window at high concentrations. GelMA remained at low *Oh* (typically <0.1) with ${L_{ar}}\,$ between ∼1.9 and ∼3.8, reflecting long ligaments and a tendency toward satellite formation when the inertial impulse was high.

Adding T20 shifted all bioinks slightly to the right in *Oh* and produced small increases in ${L_{{\mathrm{ar}}}}$ for the energy-limited systems, consistent with easier detachment at constant actuation. None of the bioinks entered the ‘ligament-breakup’ zone; the closest were low-*Oh* fibrinogen and the most dilute Matrigel, supporting operation at a moderate frequency and impulse to avoid over-stretching.

The *We–Fr* plane links jet energetics to impact outcomes (figure 8(B)). For each bioink, increasing concentration lowered both *We* and *Fr*, meaning less inertial energy per drop and reduced impact severity. T20 increased *We* at essentially unchanged *Fr*, consistent with a surface-tension decrease at similar jet velocity. Fibrinogen remained at comparatively high *We* and *Fr* (for example, *We* lies within the range 105.4–96.0 and *Fr* 96.3–88.8 as concentration increased from 5 to 20 mg ml^−1^), placing it near the bouncing-to-corona corridor, where satellites are likely if the impulse is not moderated. Collagen type I traversed from an inertio-capillary region at 1 mg ml^−1^ (*We*= 23.8, *Fr =* 62.4) to an energy-limited corner at 7 mg ml^−1^ (*We =* 0.90, *Fr* = 26.7), explaining the shorter ligaments and occasional incomplete pinch-off at high solids unless velocity or dwell is increased. Matrigel followed a similar but milder trend (*We* varied from 65.6 to 9.31 and *Fr* from 86.2 to 48.1 over a concentration range of 0.1%–10%), while alginate stayed in a middle band (*We* varies from 65.7 to 16.5 and *Fr* from 91.1 to 58.3 over a concentration range of 0.05%–0.5%), supporting stable impact without splashing. Agarose rapidly entered the low-*We*, low-*Fr* corner at high concentrations, indicating a strongly energy-limited regime where higher velocity or lower concentration is required for clean detachment. GelMA remained at moderate-to-high *We* and *Fr* across its range, placing it closer to the satellite-prone corridor at low concentrations and requiring moderation of impulse for stable single-drop formation. Practically, T20 moves all bioinks to higher *We* at the same *Fr*, improving detachment for energy-limited cases but nudging fibrinogen closer to splash-onset unless the impulse is trimmed.

The *We-Oh* map separates impact-driven from capillary-driven jetting (figure 8(C)). Collagen type I shifted strongly to the right with concentration (*Oh* from 0.051 to 5.81 in the absence of T20 and from 0.064 to 6.74 in the presence of T20), placing the concentrated samples in a *η*-dominated zone, where additional impulse is needed for clean detachment. Fibrinogen remained at very low *Oh* (from 0.0086 to 0.0127), confirming an inertio-capillary regime that explains its satellite tendency when *We* is high. Matrigel progressed from thin-liquid behavior at low concentrations to more viscous response at high concentrations (*Oh* from 0.0127 to 0.206 without surfactant and from 0.0156 to 0.248 with T20). Alginate stayed within a capillarity-driven, printable band (*Oh* from 0.0093 to 0.070 without T20 and from 0.0123 to 0.0915 with T20). Agarose moved deep into the high-*Oh* region even at moderate concentrations, explaining its short ligaments and frequent energy-limited detachment at high solids. GelMA remained at low *Oh* across all concentrations, placing it in the satellite-prone regime unless *We* is carefully moderated. If a condition falls to the right of the printable band (i.e., at higher *Oh* due to greater *η*), detachment can be restored by slightly increasing inertial drive (higher jet velocity/impulse, raising *We*) or by lowering *η* (e.g., a small decrease in concentration). Conversely, when *Oh* is very low (inviscid regime), the system is prone to satellites; this can be suppressed by modestly reducing the impulse (i.e., shortening dwell or lowering velocity/back-pressure) to bring *We* down.

Figure 8(D) highlights the two dominant failure modes: insufficient energy at low *We* and *Re*, and satellite droplets at high *We* and *Re*. Fibrinogen combined high *Re* from 1192 to 773 with high *We* from 105 to 134, clustering near the satellite domain and calling for moderation of dwell or pressure when T20 was present. Collagen type I moved toward the opposite corner as concentration increased (*Re* decreased from 95 to 0.16 with *We* decreased from 23.8 to 0.90), which explains the occasional long filaments and delayed pinch-off at high solids unless the jet velocity was raised. Matrigel spanned an intermediate region (*Re* decreased from 637 to 15 and *We* decreased from 65.6 to 9.31), and T20 raised *We* at each concentration (e.g., from 65.6 to 98.4 at 0.1%, and from 9.31 to 14.5 at 10%) similar to *Re* so that diluted formulations approached the printable corridor while the most concentrated ones still required more impulse. Alginate occupied the broadest printable window (*Re* decreased from 871 to 58 and *We* decreased from 65.7 to 16.5), and T20 raised *We* at each concentration (i.e., from 65.7 to 115 at 0.05% and from 16.5 to 29.6 at 0.5%) similarly *Re* without leaving the printable window. Agarose clustered in the energy-limited corner at high concentrations (low *Re*, low *We*), requiring increased velocity for clean detachment. GelMA clustered near the high-*We*, high-*Re* corridor at low concentration, explaining its higher satellite propensity unless impulse was reduced. In short, energy-limited bioinks need higher *We* or lower *Oh*; satellite-prone bioinks need slightly lower *We* or reduced frequency.

The *Oh–Re* map consolidates the guidance by separating an energy-limited zone at high *Oh* and low *Re*, a printable band in the middle, and a satellite-prone zone at low *Oh* and high *Re* (figure 8(e)). Fibrinogen sat at low *Oh* from 0.0086 to 0.0127 and high *Re* from 1192 to 773, with T20 increasing *Oh* slightly (from 0.0097 to 0.0134); printability is high, but satellite risk rises if the impulse is excessive. Collagen type I traversed low-to-very-low *Re* from 95 to 0.16 with *Oh* from 0.051 to above 1, and with T20 to above 6; concentrated samples were energy-limited and benefited from either dilution or higher velocity. Matrigel moved from the printable band at low- to mid-concentrations toward the energy-limited corner at the highest solids, while alginate remained in the printable band across its range with a comfortable margin. Agarose resided almost entirely in the energy-limited zone at high concentrations, whereas GelMA remained in the satellite-prone zone at low concentrations.

Taken together, the five maps show that alginate was the most robust and concentration-tolerant bioink. Matrigel was printable at low- to mid-concentrations but becomes energy-limited at the highest concentrations. Fibrinogen was printable but satellite-prone because of its low *Oh* and high *We*, and collagen type I became *η*-limited at high concentration unless the jetting impulse was increased or *η* was reduced. Agarose exhibited the narrowest printable window, rapidly becoming energy-limited at high concentrations, whereas GelMA was highly sensitive to impulse because of its persistently low *Oh* and long ligaments. T20 lowered *σ* and therefore increased *We*, while also increasing *Oh*. It assisted energy-limited bioinks, such as collagen type I, alginate, and diluted Matrigel, by easing detachment, but it could intensify satellite tendency for fibrinogen unless actuation was moderated. Practically, selecting dwell time, pressure, and frequency to place each bioink in the mid-band of the *Oh–Re* and *We–Re* maps, together with keeping ${L_{ar}}\,$ between about 1 and 3, yielded stable single-drops with minimal satellite formation for a 250 *µ*m nozzle.

**Figure 9.**
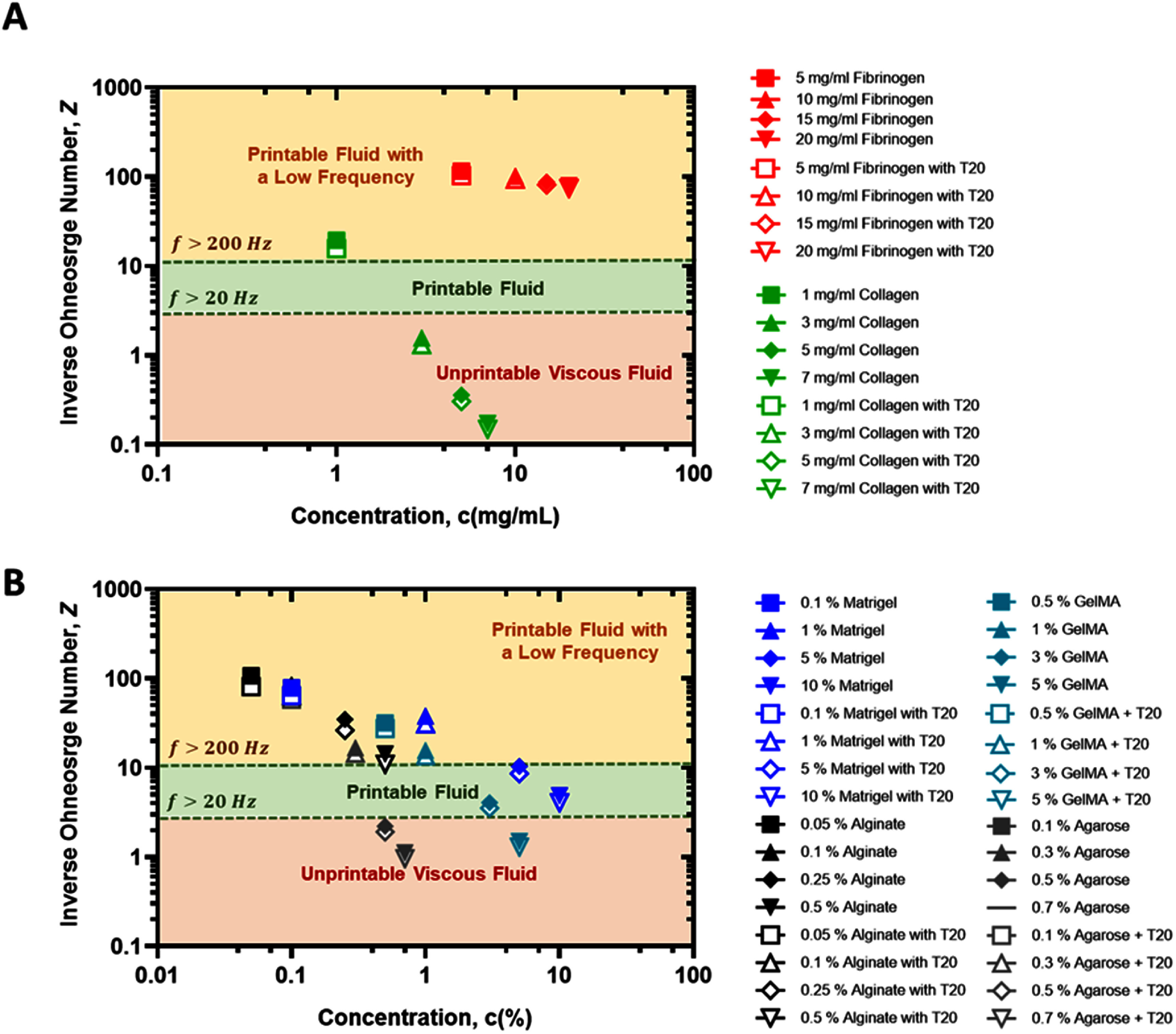
(A) *Oh*-frequency (*f*) versus concentration map for collagen and fibrinogen bioinks. Shaded regions summarize operational outcomes: ‘Printable fluid’ (20 Hz < *f*< 200 Hz), ‘Printable fluid with a low frequency’ (*f* > 200 Hz), and ‘Unprintable viscous fluid’ (*f* < 20 Hz). Each marker corresponds to formulations with/without T20. (B) The *Oh-f* versus concentration map for Matrigel and alginate bioinks. Symbols denote different formulations, highlighting the influence of viscosity and T20 addition on attainable printing frequencies.

To place our results in a practical context, we next considered the operating limits on jetting frequency. For reliable droplet generation, the frequency must be slow enough to allow a ligament to thin and pinch off completely before the next cycle [61, 78, 79]. This timescale was set mainly by *σ* and *η*: lower *σ* (as with T20) makes ligaments recoil faster and allows slightly higher frequencies, while higher *η* slows pinch-off and reduces the usable range [28, 80, 81]. With a 250 *µ*m nozzle and the measured bioink properties, the characteristic pinch-off time is only a few tenths of a millisecond, which translates to workable jetting frequencies in the range of tens to a few hundred hertz. The jetting frequency (*f*) must be slow enough for a ligament to thin and pinch off within one cycle. A useful scaling is that the pinch-off time scales with the capillary time (*τc*) multiplied by a viscous factor that grows with *Oh*. Lowering *σ* with T20 shortens *τc* and raises *f*_max_ slightly, whereas increasing *η* raises *Oh* and lowers *f*_max_. With a 250 *µ*m nozzle and measured properties here, *τc* is on the order of a few tenths of a millisecond, so frequencies in the tens to a few hundreds of Hertz are appropriate, consistent with the dashed bands in figure 9.

Across 5–20 mg ml^−1^, very low *Oh* values keep the fluid in an inertio-capillary regime for fibrinogen (figure 9(A)). The map places these points in the high-*Z* zone marked ‘printable fluid with a low frequency,’ which recommends operating in the lower part of the range, about 20–100 Hertz (Hz), to limit satellites. With T20, *Oh* increases slightly and *Z* decreases, so the allowable frequency does not widen significantly; keeping *f* near the lower end of the band remains advisable. For collagen type I, concentration moves from high-*Z* toward viscous-limited behavior (figure 9(A)). At 1 mg ml^−1^ (*Oh* ≈ 0.05, *Z* ≈ 20) clean ejection is feasible at higher frequencies, about 100–200 Hz. At 3 mg ml (*Oh* ≈ 0.64, *Z* ≈ 1.6), the map falls squarely in the printable window where 50–150 Hz is appropriate. At 5–7 mg ml^−1^ (*Oh* ≈ 2.8–5.8, *Z* < 1) the ligament thins slowly, and detachment becomes energy-limited; frequencies should be reduced to about 20–50 Hz unless the jet velocity is increased to lift *We*. Adding T20 raises *Oh* further, so the recommended frequency windows are unchanged or slightly narrower at the highest concentrations.

For Matrigel, over 0.1% to 10%, the bioink transitioned from high-*Z* at low concentration to the printable band at the high end (figure 9(B)). Low concentrations were printable but we preferred lower frequency to avoid satellites, roughly 20–100 Hz, while the 10% condition supported 50–200 Hz. T20 nudges the high-concentration points toward the center of the printable window; frequency guidance stays within the same bands, with a modest preference for the midrange. For alginate, from 0.05% to 0.5%, *Oh* rose only mildly and Z remained high, so the bioink was printable across the whole range but benefits from moderate frequencies, typically 50–200 Hz, to suppress satellites (figure 9(B)). For agarose, increasing concentration rapidly reduced *Z* from the printable band toward the unprintable viscous regime (figure 9(B)). At 0.1%–0.3%, operation was feasible at ∼50–150 Hz, whereas at 0.5%–0.7% (*Z*< 1) frequencies should be reduced to ∼20–50 Hz unless jet velocity is increased to overcome energy-limited detachment. For GelMA, *Z* remained relatively high across 0.5%–5%, placing most conditions in the printable band but closer to the satellite-prone region. Accordingly, lower-to-mid frequencies (∼20–100 Hz) were preferable to avoid excessive ligament elongation and satellite formation. With T20, *Oh* increased and *Z* approached the upper edge of the printable band at the highest concentration; recommended frequencies remained in the same 50–200 Hz window.

Overall, frequency selection followed the same physics as the *Z* map. High-*Z*, low-*Oh* bioinks, such as fibrinogen and low concentration alginate, were printable but preferred the lower- to mid-frequency range to avoid satellites. Collagen type I demanded progressively lower frequencies as concentration increased, unless *We* was raised. Matrigel moved from low-frequency preference at low concentrations to a broader frequency window at high solids. Agarose exhibited the narrowest usable frequency window at high concentrations because of rapid *Z* reduction, whereas GelMA remained sensitive to excessive frequency because of its persistently high *Z* and long ligaments. T20 shortened *τc* but increased *Oh*; in practice it did not enlarge the usable frequency range, and the safest strategy was to keep *f* within the tens to low hundreds of Hz while tuning dwell time and velocity to stay within the printable window.

### 3.4. Wettability characteristics

**Figure 10.**
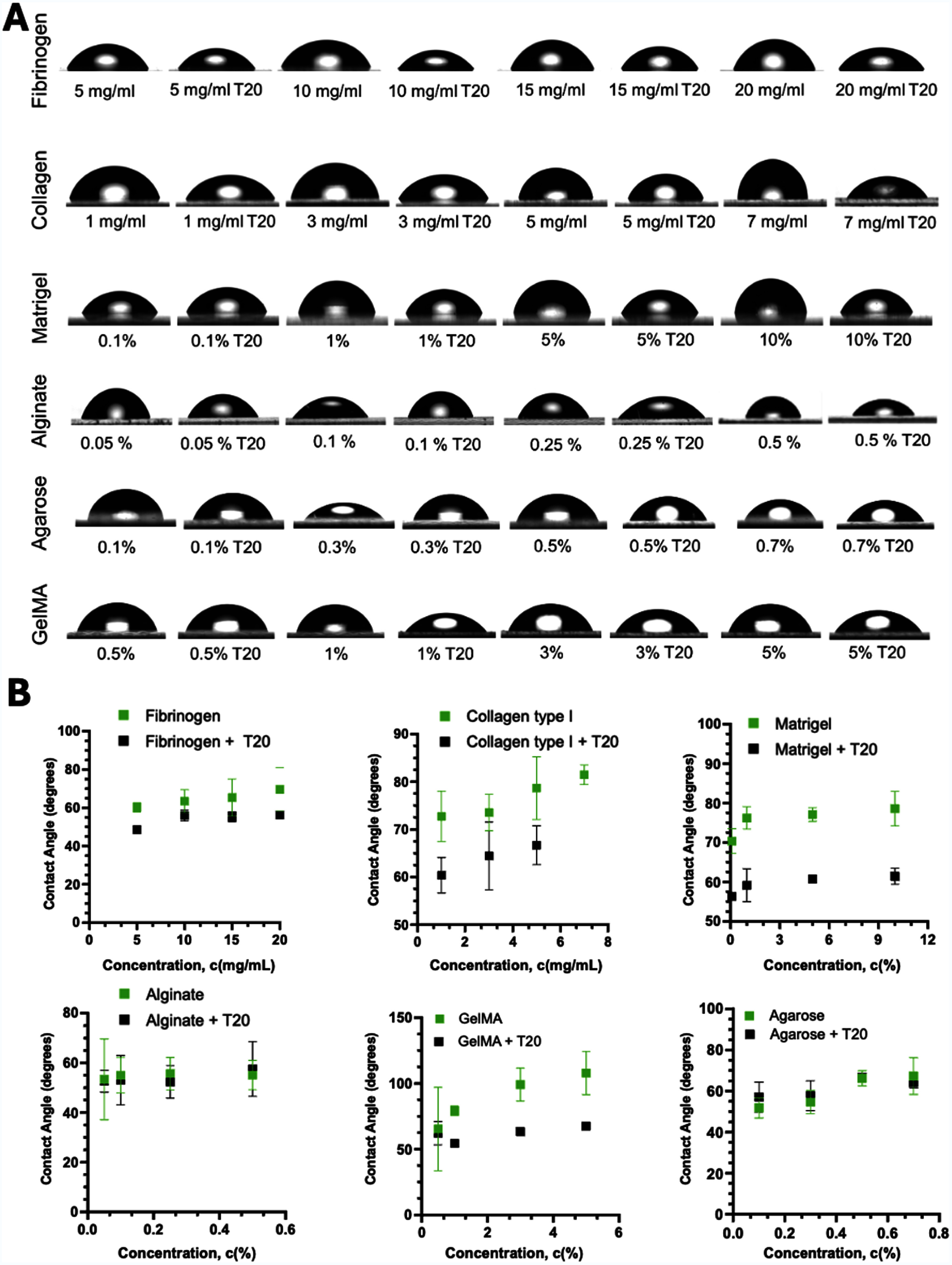
Contact angle measurements of bioinks used in MBB. (A) Representative droplet images show the effect of bioink concentrations and the addition of T20 on wettability. (B) Quantitative analysis confirms the trend, with T20 containing samples consistently displaying significantly lower contact angles compared to their untreated counterparts. Error bars represent mean ± SD (*n* = 3).

Contact angle analysis revealed distinct wettability profiles for the four tested bioinks (figure 10). For collagen type I, increasing the concentration from 1 to 7 mg ml^−1^ elevated the contact angle from ∼45° to ∼65°, indicating reduced spreading and enhanced hydrophobicity with higher macromolecular content. A similar trend was observed for fibrinogen, where contact angles increased from ∼40° at 5 mg ml^−1^ to ∼70° at 20 mg ml^−1^, reflecting a similar reduction in substrate wettability. Matrigel displayed intrinsically higher contact angles compared to other bioink solutions, exceeding 70° at concentrations of 5%–10%, consistent with its viscous, basement membrane-like composition and resulting low surface wetting (figure 10(A)). In contrast, alginate maintained relatively lower contact angles (35°–55° in the 0.05%–0.5% range), suggesting better wettability at comparable concentrations. Agarose exhibited a moderate but systematic increase in contact angle from ∼50° at 0.1% to ∼65° at 0.7%, indicating reduced spreading with increasing polymer concentration and progressive surface stiffening. GelMA showed the highest absolute contact angles among all tested bioinks, increasing from ∼65° at 0.5% to >100° at 5%, reflecting its highly hydrophobic pre-crosslinking surface and limited intrinsic wettability.

Across all bioinks, the addition of T20 reduced contact angles by ∼10°–20°, with the most pronounced effects at lower concentrations (e.g., collagen type I (1 mg ml^−1^) decreased from ∼45° to ∼30°, alginate (0.05%) decreased from ∼40° to ∼25°) (figure 10(B)). At higher concentrations (⩾10 mg ml^−1^ fibrinogen or collagen type I or ⩾10% Matrigel), contact angles remained elevated (>60°), but T20 still provided a measurable reduction of ∼8°–12°. For agarose and GelMA, T20 likewise reduced contact angles by ∼10°–25° across all concentrations, shifting both materials toward improved wettability and more reproducible spreading behavior. These results highlight that while protein-rich and ECM-based bioinks exhibit inherently poor wettability, the incorporation of T20 markedly improved droplet spreading and stability across all formulations, thereby enhancing printability in MBB.

### 3.5. Cell viability

**Figure 11.**
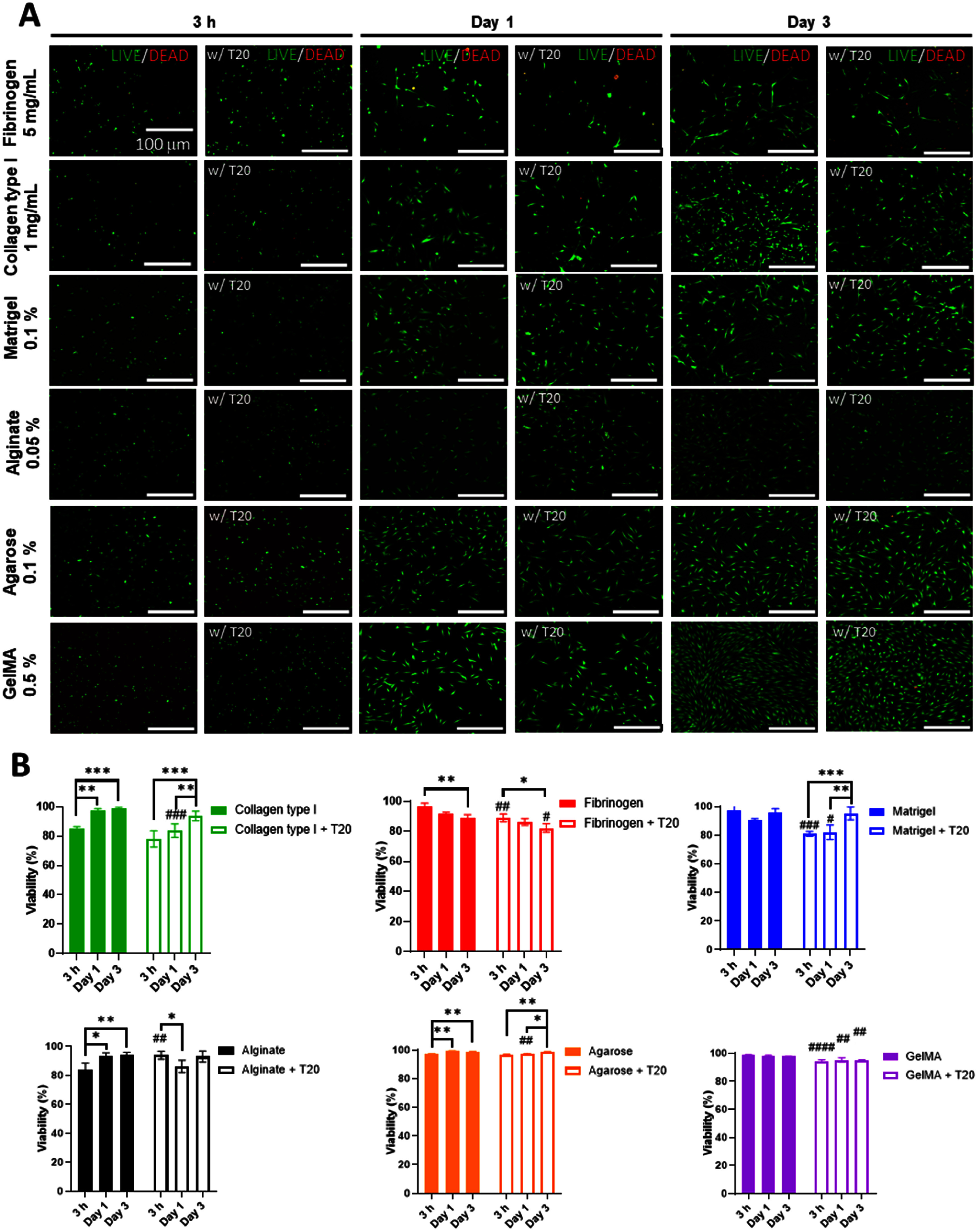
(A) Representative LIVE/DEAD fluorescence images (Calcein AM, green; ethidium homodimer-1, red) of cells bioprinted in four bioinks; fibrinogen, collagen type I, Matrigel, and alginate with and without T20 at a cell density of 0.5 × 10^6^ at 3 h, Day 1 and Day 3. (B) Quantification of post-bioprinting viability for the same conditions (Data were presented as mean ± SD; n = 3; ^*^P < 0.05, ^**^P < 0.01, ^***^P < 0.001; ^#^P < 0.05, ^##^P < 0.01, ^###^P < 0.001 (^#^ indicates significance with respect to the control (without T20) of the same group).

Cellular constructs were bioprinted at a constant droplet volume (∼60 nl) and fixed cell density (0.5 × 10^6^cells ml^−1^) for all bioinks and their corresponding T20 formulations. Cell viability was evaluated using the LIVE/DEAD assay at 3 h, Day 1, and Day 3 post bioprinting (figure 11(A)). When the 3 h time-point was examined, the highest viability was observed for fibrinogen (∼98%), while the lowest viability was observed for collagen type I with T20 (∼74%). At Day 1, the highest viability was observed for collagen type I (∼97%), while the lowest was observed for collagen type I with T20 (∼78%), whereas at Day 3, the collagen type I group maintained the highest viability (99.43%), and the group with the lowest viability (∼81%) was fibrinogen with T20. Among the T20-added groups, fibrinogen showed decreased viability at different time points, from ∼93% to ∼81%, while in the other T20-added groups, viability was above 70% at the 3 h and Day 1 time-points, and above 80% at Day 3 (figure 11(B)). Alginate, agarose, and GelMA exhibited consistently high viability across all time points, with values generally remaining above 85% at Day 1 and above 90% at Day 3, indicating that these matrices provide a more stable and protective microenvironment during and after jetting. Notably, agarose and GelMA showed minimal sensitivity to T20 addition, with no systematic decrease in viability across the three time-points, consistent with their mechanically stable and physically crosslinked networks. The lower viability observed in fibrinogen might be related to the limited matrix stability as only post-bioprinting enzymatic conversion to fibrin provides mechanical support. In contrast, collagen type I, alginate, agarose, and GelMA provide immediate physical confinement upon deposition, which likely contributes to their improved short- and long-term cell survival.

In addition to viability, cytoskeletal organization and cell morphology within the bioprinted constructs were evaluated by Alexa Fluor 568-conjugated phalloidin and DAPI staining (figure S1). Across all bioink formulations, NHDFs exhibited well-developed actin filaments and elongated, spindle-like morphologies, indicative of preserved cytoskeletal integrity after jetting. Phalloidin staining revealed continuous F-actin stress fibers distributed along the cell body, while DAPI counterstaining confirmed uniform nuclear morphology without signs of fragmentation or condensation. Notably, cells in mechanically stable matrices such as alginate, agarose, and GelMA displayed more homogeneous spreading and cytoskeletal alignment, consistent with their high viability and favorable microenvironment. These observations corroborated with LIVE/DEAD assay results and demonstrated that MBB preserved not only cell survival but also cytoskeletal organization and normal NHDF morphology following droplet-based deposition.

Collectively, these results indicate that MBB reliably enables droplet-based deposition of cell-laden bioinks with high short- and intermediate-term viability across diverse hydrogel systems. The impact of T20 was bioink-dependent: while it was generally compatible with alginate, Matrigel, collagen type I, agarose and GelMA, its prolonged presence in fibrinogen-based formulations was associated with a gradual reduction in viability over extended culture periods. These findings underscore the necessity for careful surfactant optimization tailored to both cell type and bioink composition when developing bioprinted tissues.

### 3.6. Power-law fitting and FE simulations

Rheological characterization of all bioinks demonstrated pronounced shear-thinning behavior, with power-law indices (*n*) ranging from 0.15 < *n* ⩽ 1. For all formulations, both with and without the surfactant Tween 20 (T20), an increase in bioink concentration generally corresponded to a decrease in the flow behavior index (*n*) and an increase in the consistency index (*K*) (tables S7 and S8). Deviations from this trend were observed for alginate and GelMA formulations in the absence of T20, and for fibrinogen, Matrigel, alginate, and GelMA when T20 was included. These variations are attributed to batch-to-batch differences in bioink preparation, experimental noise during rheological measurements, and uncertainties associated with curve fitting of noisy datasets. The power-law indices for GelMA and alginate remained close to unity at all concentrations, regardless of surfactant addition, indicating near-Newtonian flow behavior. Similar behavior was observed for fibrinogen and Matrigel in the presence of T20. Such near-Newtonian properties may account for the subtle irregularities observed in the fitted rheological parameters.

The viscosity model further suggests that, within the nozzle, the velocity, shear rate, and apparent viscosity are nonlinearly distributed. The no-slip boundary condition at the nozzle wall results in zero local velocity, a maximum shear rate, and a minimum apparent viscosity, whereas the nozzle centerline exhibits the maximum velocity, zero shear rate, and an apparent viscosity singularity due to the vanishing shear rate.

Power-law parameterization of the experimental data enabled subsequent FE simulations of flow behavior during the bioprinting process. These simulations quantified local shear rates and apparent viscosities within the nozzle, particularly near the wall region where cells experience the highest deformation. Due to the viscosity singularity at zero shear rate along the nozzle centerline, numerical regularization was applied. COMSOL Multiphysics® addresses this by defining a small finite minimum shear rate, effectively generating a high but finite apparent viscosity at the centerline to ensure numerical stability. The FE-derived shear rates and apparent viscosities for each bioink are summarized in tables S7 and S8, showing wall shear rates on the order of 10^4^–10^5^ s^−1^, and apparent viscosities range from 0.08 to 7.6 mPa s.

The FE-generated cross-sectional profiles of velocity and shear rate further illustrate the influence of bioink rheology on nozzle flow dynamics. For 0.1% Matrigel, which exhibited Newtonian behavior with *n* = 1, the velocity and shear rate distributions were parabolic and linear, respectively, consistent with classical Poiseuille flow (figures S2(A) and (B)). In contrast, 0.7% agarose containing T20 (a shear-thinning bioink with *n* = 0.22) exhibited distinctly non-linear velocity and shear rate profiles across the nozzle cross-section (figures S2(C) and (D)). These results underscore the necessity of accurate rheological model fitting and FE-based flow simulations for predicting local mechanical environments relevant to cell viability and deposition fidelity during bioprinting.

## 4. Conclusions and future outlook

This study provides a coherent, physics-informed framework for MBB that links measurable properties to jetting outcomes through a small set of dimensionless groups. By mapping six representative bioinks, fibrinogen, collagen type I, Matrigel, and alginate, with and without a surfactant (T20), onto printability spaces spanned by *Oh, We, Re, Fr,* *Z*, and the ${L_{{\mathrm{ar}}}}$, we showed how concentration and interfacial tuning reorganize the balance of inertia, viscosity, capillarity, and gravity. Under fixed actuation, concentration mainly changes *η* and thus ligament damping and pinch-off timing, while T20 mainly lowers *σ* to improve repeatability and suppress satellite formation without shifting mean volume. Cell loading up to three million cells per milliliter preserved mean ligament volume and high viability, indicating that the identified operating windows were compatible with cell viability. The framework offers a practical way to place any new formulation on a printability map and choose settings that favor stable single-drop operation.

In this study, a single nozzle diameter was used to ensure consistent droplet formation dynamics and eliminate confounding geometric factors. While this approach enabled clearer interpretation of printability regimes, it inherently limits generalizability across different nozzle geometries. We acknowledge this constraint and suggest that future studies expand the framework by incorporating various nozzle sizes to assess the scale dependence of ligament behavior and droplet stability.

The same toolkit can be applied directly to new cell-laden bioinks, alternative nozzle diameters (via rescaling in *Oh, We, Re, Fr, and Z*), and other DBB platforms. Rheological behavior was characterized within each bioink group across its relevant printable concentration range, rather than inter-bioink comparisons due to differences in bioinks’ molecular weight, polymer structure, and physiologically relevant concentration windows. Since each bioink exhibits different printable ranges, comparing their viscosities at different concentrations may not yield universally valid hierarchies. Therefore, rheological assessments were interpreted within bioink-specific trends rather than across bioinks.

The printability maps presented in this study are intended as a semi-quantitative framework that provides physically interpretable classification of droplet formation and jetting regimes. The zoning approach is based on experimentally observed transitions in ligament formation, breakup behavior, and droplet stability, rather than statistically deterministic predictions. While this heuristic classification enables robust comparison across bioinks with distinct rheological and gelation characteristics, it does not constitute a fully predictive model. Future studies may extend this framework by integrating expanded datasets, probabilistic regime boundaries, or data-driven modeling approaches to improve predictive capability.

While clean pinch-off and minimal satellite formation are essential criteria for evaluating printability in MBB, they primarily reflect the jetting phase and do not fully account for the subsequent stages of droplet landing, wetting, coalescence, and structural buildup. For example, even droplets with ideal formation dynamics may result in poor geometric fidelity if they exhibit excessive spreading or rebound upon impact, or if they fail to coalesce uniformly with adjacent droplets. In addition, substrate properties, environmental conditions, and gelation kinetics can significantly affect final construct morphology. Therefore, achieving satellite-free droplet generation should be considered a necessary but not sufficient condition for ensuring robust structural fidelity in MBB. Future frameworks may benefit from integrating droplet formation dynamics with post-landing behavior to predict and control bioprinting outcomes more reliably

Immediate extensions include expanding to higher cell volume fractions and diverse cell types, where crowding, yield-stress onsets, and interfacial adsorption may shift *Oh* and *We*; quantifying temperature sensitivity for thermo-responsive systems (collagen fibrillogenesis, Matrigel gelation and agarose thermal gelation), and substrate temperature; and integrating crosslinking dynamics (enzymatic for fibrin, ionic for alginate, thermal for collagen, Matrigel, and agarose, and photo-crosslinking for GelMA) by comparing gelation time scales with capillary and flight times to enable in-flight or on-impact curing. Complementary measurements of extensional and interfacial rheology should refine ${L_{{\mathrm{ar}}}}$ -based criteria for satellite suppression, while studies of nozzle wetting and antifouling coatings can reduce drift in *σ* and mitigate clogging. Finally, coupling these maps with real-time imaging and simple adaptive control or lightweight physics-informed machine learning can close the loop on the bioprinter, automatically steering a formulation toward its optimal *Oh*–*Re–We*–${L_{{\mathrm{ar}}}}$ region across protein- and polysaccharide-based, and photocurable bioinks such as collagen, alginate, agarose, and GelMA. By grounding MBB decisions in first-principles metrics, this approach shortens optimization cycles, improves reproducibility, and broadens the palette of constructs achievable in future bioprinting applications.

## Data Availability

All data that support the findings of this study are included within the article (and any supplementary files). Droplet Generation available at https://doi.org/10.1088/1758-5090/ae54d1/data1. Droplet Line Formation available at https://doi.org/10.1088/1758-5090/ae54d1/data2. Patterning Demonstration available at https://doi.org/10.1088/1758-5090/ae54d1/data3. Supplementary Materials available at https://doi.org/10.1088/1758-5090/ae54d1/data4.
